# Co-Expression Network Analysis of Micro-RNAs and Proteins in the Alzheimer’s Brain: A Systematic Review of Studies in the Last 10 Years

**DOI:** 10.3390/cells10123479

**Published:** 2021-12-09

**Authors:** Rachel Tasker, Joseph Rowlands, Zubair Ahmed, Valentina Di Pietro

**Affiliations:** 1 Institute of Inflammation and Ageing, University of Birmingham, Edgbaston, Birmingham B15 2TT, UK; rxt872@student.bham.ac.uk (R.T.); joe.j.rowlands747@gmail.com (J.R.); z.ahmed.1@bham.ac.uk (Z.A.); 2 Surgical Reconstruction and Microbiology Research Centre, National Institute for Health Research, Queen Elizabeth Hospital, Birmingham B15 2TH, UK; 3 Centre for Trauma Sciences Research, University of Birmingham, Edgbaston, Birmingham B15 2TT, UK

**Keywords:** miRNA, Alzheimer’s disease, bioinformatics, pathway analysis, CLOCK proteins

## Abstract

MicroRNAs (miRNAs) are small non-coding nucleic acids that can regulate post-transcriptional gene expression by binding to complementary sequences of target mRNA. Evidence showed that dysregulated miRNA expression may be associated with neurological conditions such as Alzheimer’s disease (AD). In this study, we combined the results of two independent systematic reviews aiming to unveil the co-expression network of miRNAs and proteins in brain tissues of AD patients. Twenty-eight studies including a total of 113 differentially expressed miRNAs (53 of them validated by qRT-PCR), and 26 studies including a total of 196 proteins differentially expressed in AD brains compared to healthy age matched controls were selected. Pathways analyses were performed on the results of the two reviews and 39 common pathways were identified. A further bioinformatic analysis was performed to match miRNA and protein targets with an inverse relation. This revealed 249 inverse relationships in 28 common pathways, representing new potential targets for therapeutic intervention. A meta-analysis, whenever possible, revealed miR-132-3p and miR-16 as consistently downregulated in late-stage AD across the literature. While no inverse relationships between miR-132-3p and proteins were found, miR-16′s inverse relationship with CLOCK proteins in the circadian rhythm pathway is discussed and therapeutic targets are proposed. The most significant miRNA dysregulated pathway highlighted in this review was the hippo signaling pathway with *p* = 1.66 × 10^−9^. Our study has revealed new mechanisms for AD pathogenesis and this is discussed along with opportunities to develop novel miRNA-based drugs to target these pathways.

## 1. Introduction

Alzheimer’s disease (AD) is the most common form of dementia. In 2019, there were 5.8 million Americans living with AD [1]. This number is projected to rise to 13.8 million Americans in 2050 and has a worldwide prevalence of 131.5 million [2]. While deaths due to many common age-associated illnesses such as heart disease are declining each year, deaths due to AD increased 146.2% from 2000 to 2018 [3]. Furthermore, the socioeconomic disease burden is huge, 305 billion USD is spent annually on long term and hospice care for AD patients [4]. Supporting patients incurs 18.6 billion care hours from 16 million family members and care workers estimated a 244 billion USD annual loss, not to mention the emotional distress, fatigue, and negative impact on long term health for those carers [5,6]. The risk of developing Alzheimer’s increases with age affecting 3% of 65–74 year olds, 17% of 75–84 year olds, and 35–50% of people over the age of 85 [7]. Two-thirds of AD patients are women—in part due to longer life expectancy but also due to increased genetic risk [7,8]. The disease disproportionately affects those from underprivileged, minority groups with Hispanics and African Americans 1.5 times more likely to develop AD [9,10]. Poor education, co-incidence of cardiovascular disease, lack of exercise, social isolation, and repetitive concussions all increase likelihood of developing the disease [11,12,13,14]. For these reasons, the WHO has defined AD as a world health priority.

The hallmarks of AD are the accumulation of extracellular β-amyloid (Aβ) plaques and intracellular neurofibrillary tangles (NFT) of hyperphosphorylated Tau protein [15,16]. These contribute to progressive, irreversible neurodegeneration in the form of synaptic loss and neuronal apoptosis, along with psychiatric symptoms including memory loss, depression, and anxiety that greatly reduce the independence of an individual and eventually culminate in death. Although the mechanisms contributing to AD pathology are diverse, historically, the research focus has surrounded these two hallmarks which fail to generate a holistic etiology and has hindered meaningful progress in the development of treatments showing the need for wider studies and explanations [16].

Despite the FDA approving the first Alzheimer’s medication recently, which works by clearing out amyloid plaques, it was criticized that the effective reduction of plaque load has not produced commensurate clinical benefits [17]. Currently, there are no disease-modifying therapies that can prevent or slow the progression of AD, and so current treatments focus on the management of symptoms. The lack of success with these drugs may be due to administration late in AD progression, and when neurodegeneration is already widespread and irreversible but may also reflect the centrality of Aβ and Tau independent pathologies [18]. Together, these findings highlight the desperate need to expand the search for drug targets to treat AD. Multiple genetic defects are linked to the development of AD and in 95% of cases the mutations are sporadic. However, homozygous inheritance of Apolipoprotein E 4 (ApoE4) confers a 10–15-fold increase in risk of developing AD due to its weak binding interaction with Aβ and competing with Aβ for binding to the LRP-1 receptor, resulting in reduced clearance [19].

Emerging evidence also suggests that epigenetic mechanisms can contribute to the development of the pathology. Gene–enviroment interactions, nutrients, and toxins can activate or silence gene expression without any alteration on the genome sequence, influencing the phenotype through DNA methylation, histone modification, and microRNA expression. MicroRNAs (miRNAs, miRs) are small non-coding, single-stranded nucleic acids 18–22 nucleotides long. They regulate the expression of more than 60% of coding genes of the human genome by binding to specific sites within the 3′UTR of the target mRNA and leading to mRNA degradation or translation inhibition. Recent studies demonstratethat miRNAs may also promote gene expression by binding the 5′UTR or other parts of mRNA [20,21,22]. Therefore, miRNAs may be implicated in several biological processes, such as proliferation, differentiation [23], development and organization of the CNS, synaptic plasticity, and memory formation [24]. It is therefore unsurprising that miRNA dysregulation has been observed in a plethora of neurodevelopmental, neuropsychiatric, and neurodegenerative disorders from autism to AD [25]. In addition, miRNAs are aberrantly expressed in the AD brain; however, functional assays tend to limit their focus to relating findings to the Aβ or Tau hypothesis [26,27,28,29]. For example, miR-339-5p, miR-107, and miR-29c bind to the 3′ UTR of BACE1 to negatively regulate ß-secretase transcription [30,31]. Each is downregulated in AD which promotes the amyloidogenic cleavage of the APP protein and excessive Aβ production in the AD brain. In vitro studies show that miR-106a/b, miR-17-5p, and miR-20a are able to bind to the 3′UTR of APP mRNA and suppress its transcription. Post-mortem analysis shows that miR-16, miR-152b, and miR-132-3p are upregulated in early AD and likely induce phosphorylation of Tau [32,33].

MiRNA-based therapies are fast gaining recognition as a high potential novel treatment approach due to their high specificity resulting in over 500 patents being taken out in 2018 [34]. Downregulated miRNA can be replaced with synthetic oligonucleotides coined “miRNA mimics”, and the action of over-expressed miRNA can be suppressed through the formulation of anti-sense oligonucleotides known as miRNA suppressors [35]. Furthermore, drugs modulating the activity of miRNA can be packaged into 20–10 nm lipid vesicles or nanoparticles that are under 400 Daltons to facilitate crossing of the BBB making them ideal candidates to treat neurodegenerative diseases in the CNS [36]. While there are no currently approved miRNA therapies on the market, primate trials show that miRNA therapies have high efficacy and low toxicity [37]. This gives hope for miRNA as a high potential drug candidate. As there are currently no disease-modifying therapies for AD, miRNA present an ideal new avenue for treatment research and wider research into protein regulation by miRNA in the AD brain would assist miRNA drug target selection.

To date, there has been no comprehensive review of the crosstalk between miRNA and protein expression in the AD brain. Therefore, in this study, we perform parallel systematic reviews and meta-analyses of miRNA and protein expression in the post-mortem AD brain and combine this with bioinformatic network analysis. In this way, we aim to identify new mechanistic contributions to the pathology of AD and predicted miRNA targets through their inverse expression which can be used to design powerful therapeutic tools.

## 2. Materials and Methods

### 2.1. Study Design

Both systematic reviews and meta-analysis were undertaken using the Preferred Reporting Items for Systematic Reviews and Meta-Analyses (RPISMA) guidelines [38]. The research question, “To identify the differentially expressed micoRNAs/proteins in Alzheimer’s disease” enabled the generation of focused keywords which were used to search and retrieve relevant records from three databases. Explicit inclusion and exclusion criteria were developed which were used to complete an abstract and full-text screening of the records retrieved to determine those eligible for inclusion. This was done by hand by a primary reviewer and then included articles were checked by a second reviewer (R.T., V.D.P. for miRNA study and J.R., V.D.P. for protein study). Data were extracted and a meta-analysis was performed whenever possible. The miRNAs and proteins identified through the literature search were entered into DIANA or DAVID database, respectively, for in silico bioinformatic analysis of predicted gene targets and Kyoto Encyclopedia of Genes and Genomes (KEGG) pathway identification.

#### 2.1.1. Search Strategy

The Population, Intervention, Comparison(s), and Outcome(s) (PICOs) framework for systematic reviews was utilized to define and focus the research question. This Cochrane Collaboration-recommended system ensures that the full scope of evidence is considered within defined parameters and a quantitative investigation of the results can be undertaken.

Population: Humans over the age of 60 from both sexes and all nationalities, who consensually donated their brains to brain banks for scientific research.

Intervention: Diagnosis with AD. Participants must have shown evidence of cognitive impairments during lifetime assessed by a clinician, and AD diagnosis must have been confirmed by a pathologist at autopsy by the prevalence of Aβ plaques and NFT.

Comparisons: Healthy age-matched controls that showed no evidence of cognitive impairment in lifetime and showed none of the hallmarks of AD (or any other neurodegenerative disease) in autopsy analysis.

Outcomes: The fold change in miRNA and protein expression profiles between AD patients and healthy age-matched controls of post-mortem brain tissues.

#### 2.1.2. Search Terms and Databases

In the microRNA systematic review, the following keywords were selected to represent the PICO criteria and combined with Boolean operators to generate the string search: “Alzheimer’s disease” AND “microRNA” or “miRNA” or “miR” and “expression” and “human” and “brain” or “brain parenchyma” or “brain tissue”. In the protein systematic review, the keywords applied to search the databases were “Alzheimer’s Disease”, “Proteins”, “Expression”, and “Human Brain” with the “AND” Boolean operative command between. The search was carried out in three databases, PubMed, EMBASE through Ovid, and Web of Science, in both reviews. A filter was applied to search for records between 2010 to February 2021. This was done to keep the search parameters up to date and in accordance with the recent advancements in miRNA research that forms the opposite side of this network analysis. The records retrieved were collated in Endnote 20 (Clarivate, Philadelphia, PA, USA) where they were screened for duplicates and any identified were removed. The remaining abstracts were then manually assessed for eligibility by two independent reviewers using our predefined inclusion and exclusion criteria outlined in Table 1.

#### 2.1.3. Data Extraction

Data were extracted from the final included studies and imported into Microsoft Excel (Microsoft Corporation, Redmond, WA, USA). In both reviews, the title of the article, authors, and abstract summaries were saved to identify the studies. The number, the mean age, and gender of AD patients and controls were recorded to assess population data and evaluate homogeneity for meta-analysis. Brain regions and methodology were also recorded. MiRNAs or proteins up- or down-regulated in AD brains compared to healthy age-matched controls were recorded (along with fold changes and standard deviations for microRNA review only). Where raw data were unavailable, values were estimated from graphs by copying graphs into ImageJ, Version 1.53a (NIH, Bethesda, MD, USA; https://imagej.nih.gov/ij/, last accessed 7 December 2021) and using the automated ‘grid’ plugin to estimate values on the *y*-axis.

#### 2.1.4. Quality Appraisal of Papers

The quality of records included in this review were assessed via the AXIS tool and Risk of Bias methodology. The AXIS tool was utilized to critically appraise the quality of the literature identified through the search strategy [39]. The examination framework is comprised of 20 questions that generate “yes”, “no”, or “not applicable” answers to objectively uncover bias in the population, methodology, presentation of results, and repeatability of studies. Specific questions have been developed for each section of the papers to allow a comprehensive overview of each article. Not all questions were applicable to the included papers and as a result, questions 7, 13, and 14 were removed since the included studies did not analyze response rate or perform non-responder analysis. The questions that were removed were: “(7) were measures undertaken to address and categorize non-responders”, “(13) does the response rate raise concerns about non-responder bias?”, “(14) is appropriate, was information about the non-responders described?”.

Each article was given a quality score percentage calculated by x = (number of “yes” responses/17) * 100 for the remaining questions. Studies with a quality score of x > 60% were included in the final review.

The Revised Cochrane risk-of-bias tool for randomized trials (RoB2) was used to further assess bias arising from the technical handling and scientific methodology employed in each study [40]. The tool utilizes five domains each comprised of a series of signaling questions that inform a final bias assessment of every aspect of the randomized trial. Trial design, adherence to protocols, and reporting of results are all considered as potential sources of bias. Each domain is graded “low”, “some concerns”, or “high” risk of bias that all contribute to an overall bias rating for each study. Records with high overall bias were not included in the final review.

#### 2.1.5. Meta-Analyses

Due to the homogenous nature of the data collected, miRNA reported to be differentially expressed in the brains of AD patients compared to age-matched controls a minimum of three times by different articles were deemed appropriate for meta-analysis. Only four miRNAs met these criteria and meta-analysis was performed using a random effects model of miRNA fold change data along with standard deviations. The random effects model was chosen as equal effects could not be assumed between studies. Forrest plots were constructed and a heterogeneity value was calculated for each miRNA. The between studies effect size was quantified using a heterogeneity threshold set at 50%. Values *I^2^* ≤ 50% were taken to mean statistical significance beyond chance was achieved.

None of the studies on proteins in AD met the criteria for the meta-analysis.

### 2.2. Pathway Analysis

DIANA tools miRpath v.3 (http://snf-515788.vm.okeanos.grnet.gr/is last accessed 20 August 2021), the most extensive bioinformatics tool, was used for miRNA pathway analysis [41]. The database applies algorithms to assess the biological significance of miRNA associations and predict their function via experimentally validated data in TarBase or in silico predicted targets in microT-CDS [42]. The miRNAs identified through this study were individually uploaded to DIANA and a superset of gene targets was identified by microT-CDS. Where selected genes unite on a particular KEGG pathway with (*p* < 0.05), this was noted as statistically significant and a potential role for miRNA regulation in AD were proposed.

Protein pathway analysis was done through the Database for Annotation, Visualization, and Integrated Discovery (DAVID) (https://david.ncifcrf.gov/ last accessed 20 August 2021) using the KEGG pathway maps and functional analysis. All differentially expressed (DE) proteins identified from the included studies were uploaded onto the DAVID database. Where protein abbreviations used in the included records were not recognized by the DAVID database, alternative aliases were used ensuring all proteins were identified. Pathway enrichment analysis was performed using functional annotation charts based off the EASE value. The threshold stringency settings were restricted to a minimum gene count of 3 for a pathway, and an EASE *p*-value of 0.1. This value was chosen as it would identify the most strongly enriched pathways since a perfect enrichment would be equal to 0 with reducing the chance of removing significant pathways out of the miRNA comparison analysis. A detailed description of the pathway analyses can be found in the Supplementary Materials.

### 2.3. Predicting miRNA-Protein Interactions through Inverse Relationships

The results of the studies were combined. Common KEGG pathways identified in both the miRNA and protein databases were selected for further analysis. MiRNA and proteins were individually matched to their genetic targets and a table containing ‘KEGG pathway’, ‘protein’, ‘miRNA’, and ‘*p* value’ was created. MiRNA were assumed to play an inhibitory role in the transcription of proteins. Therefore, an inverse correlation defined by an increase in protein expression AND a decrease in miRNA expression; or a decrease in protein expression AND an increase in miRNA expression; with the same gene targets within the same KEGG pathway was searched for. All inverse correlations suggest miRNA have a regulatory role of protein expression in that pathway and may be implicated in the pathogenesis of AD. These results, along with *p* values, were tabulated and the role of two significant pathways were further investigated in the discussion.

## 3. Results

### 3.1. Study Characteristics

**Figure 1:**
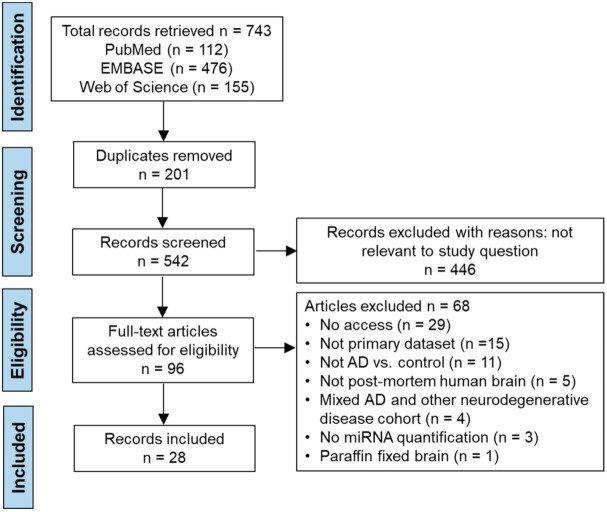
PRISMA flow chart detailing the selection and screening process utilized to retrieve the articles containing miRNAs in AD systematic review.

In the systematic review of miRNA studies, electronic searches of PubMed, EMBASE, and Web of Science generated 112, 476, and 155 records, respectively, making a total of 743 records. A total of 28 studies met all the criteria and were included in this review. During the full text assessment of studies, the greatest cause for exclusion was lack of access to the full text (*n* = 29). This was due to the high number of conference abstracts. Several studies used bio-informatics tools to reanalyze previously collected data or utilized data from inaccessible databases and were excluded from this review. Three studies only displayed qualitative miRNA expression data. Four studies were excluded because they analyzed brains with mixed neurodegenerative disease pathologies. Lastly, paraffin fixation of post-mortem tissue was deemed divergent from the standard liquid nitrogen freezing storage protocol and thus excluded from this systematic review. The selection process is summarized in the PRISMA flowchart (Figure 1).

**Figure 2:**
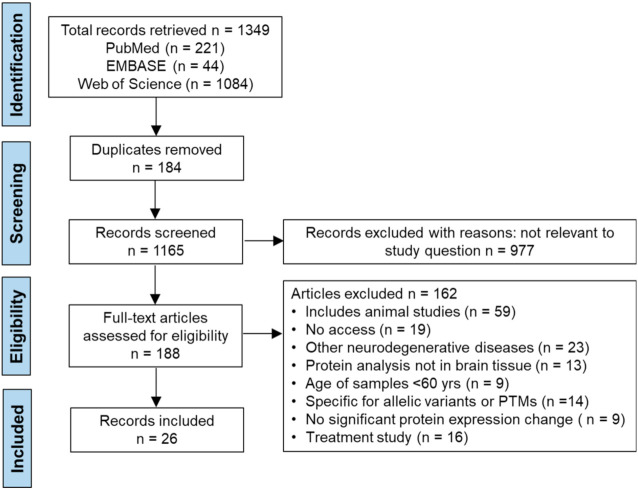
PRISMA flow chart detailing the selection and screening process utilized to retrieve the articles containing proteins in AD systematic review.

In the systematic review of protein studies, electronic searches of the databases PubMed, Embase, and Web of Science retrieved a total of 1349 citations. Using Endnote 20 (Clarivate), all citations were collated and assessed, and 184 duplicates were excluded leaving 1165 studies to be assessed for eligibility based on the title and abstracts. Following title, abstract, and full text assessment, 26 records met our inclusion criteria (Figure 2).

### 3.2. Data Extraction

Patient data were extracted from the final studies as reported in Table 2A (miRNA review) and Table 2B (protein review) to outline the scope of the research population and to inform the suitability of integrating individual studies into a meta-analysis. More details, including DE-microRNA obtained in the same studies by RNAseq, are available in the SM (Table S1).

### 3.3. AXIS Quality Appraisal

The quality of papers included in this review were assessed to determine whether participants were selected without bias, and to ensure rigorous scientific standards were met in the quality, consistency, and presentation of data. Table S2A,B show that all studies selected in miRNA and protein systematic reviews, respectively, had high quality scorings when assessed via the AXIS appraisal method. Score results were between 65 and 100% which was over the 60% threshold that we set for exclusion. The greatest reason for potential bias was the generally poor justification of the sample size in each study.

### 3.4. Risk of Bias

The RoB assessment tool was used to highlight potential bias arising from the generation and presentation of data reported in the studies included in this review (Table S3A,B). This showed that all of the studies had high overall risk of bias.

### 3.5. Meta-Analyses

**Figure 3:**
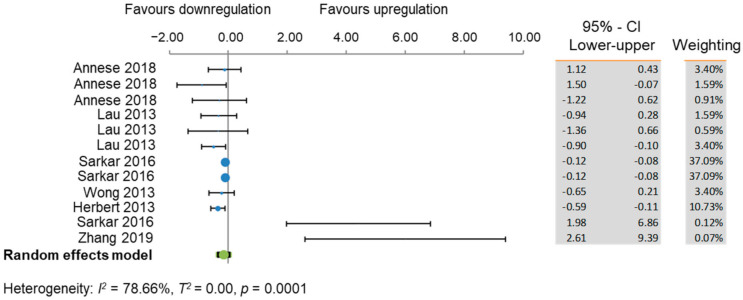
Combined meta-analysis for mRNA changes of miR-132 in AD.

**Figure 4:**
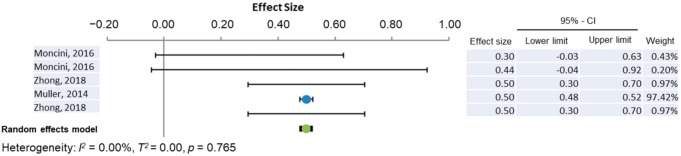
Meta-analysis of miR-16 expression in the AD brain.

Four out of the total 91 miRNAs were repeatedly observed to have aberrant expression in AD brains. These were miR-132, miR-212, miR-16, and miR-146a. Further data, including Braak stage, fold change, standard deviation, and tissue storage protocols were extracted to facilitate a better-informed comparison between the studies and to enable a meta-analysis for each miRNA. These data are presented in Table S4A–D. Studies reporting median fold change and interquartile range (IQR) instead of the mean and standard deviation were excluded as this would not have given a statistically rigorous comparison. Annese et al. [43] and Lau et al. [52] reported fold changes in log_2_(fold change) format so were converted to raw fold change by 2^{log^_2_^(fold change)}^. MiR-132-3p was the most reported miRNA across the 28 studies. Figure 3 shows that there is a general trend of downregulation of miR-132-3p in the AD post-mortem brain compared to healthy age-matched controls, whilst Annese et al. [43] (points 2 and 3) and Lau et al. [52] (point 5) cross the line of no effect at x = 1, the combined effect of a 0.31-fold change has a confidence interval of 0.18 ≤ x ≤ 0.45 and does not cross the line of no effect. This, combined with the calculated heterogeneity value of I^2^ = 0.00% which is ≤50% significance threshold, shows that there is strong evidence for the differential downregulation of miR-132-3p in AD post-mortem brains compared to healthy age-matched controls. The meta-analysis for miR-16 shows a downregulation in AD across the literature with a combined effect of 0.47-fold change and a confidence interval of 0.38 < x0.56. As the confidence interval does not cross the line of no effect at x = 1-fold change, this is significant. Furthermore, the meta-analysis generated a heterogeneity value of I^2^ = 0.00 showing that all the data are in agreement, and this is statistically significant (Figure 4). Although it appears as though there is a general trend for an miR-146a upregulated in AD brain, the confidence intervals of the combined effect (see row 8 of the forest chart and results table) cross the line of no effect at x = 1. This suggests that there is no overall change in miR-146a expression in the AD brain. The heterogeneity value calculated was I^2^ ≥ 50% significance threshold showing that the studies do not unite on any perceived effect so an upregulation could be due to chance (Figure S1).

The meta-analysis for miR-212 was unable to be completed as data were too heterogenous.

### 3.6. Pathways Analysis

Sixty-six unique KEGG pathways were identified using the DIANA-microT-CDS algorithm which shows where the 113 DE-miRNAs extracted from the 28 studies by either RT-PCR or RNAseq are predicted to interact with target genes. *p* values were generated based on the number of predicted miRNA-gene interactions. The DAVID database was used to perform enrichment analysis and pathway identification of the 196 DE-proteins. A total of 164 different KEGG pathways were identified. To determine which pathways were the most significant, functional enrichment analysis was performed using a minimum gene count ≥3 and a maximum EASE score of 0.1.

**Figure 5:**
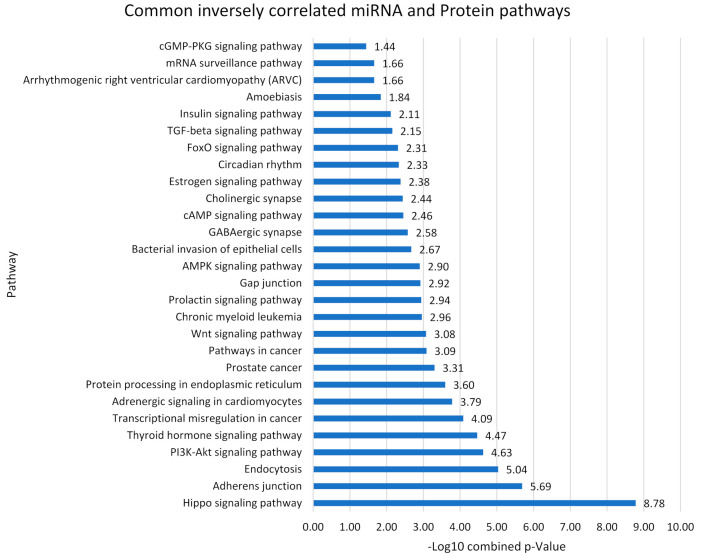
Statistical significance of the pathways that contain miRNA-protein inverse relationships. −Log10 of the combined *p*-value. Combined *p*-value calculated from miRNA *p*-value x Protein *p*-value.

Comparing the results from the DAVID and DIANA pathway databases for proteins and miRNAs, 39 common pathways were found between them. Out of the 39 common pathways identified, 28 of these included miRNAs that showed an inverse trend to the proteins involved. An overall pathway *p*-value was calculated from the miRNA statistical enrichment analysis multiplied against the Fisher’s Exact *p*-value from the protein enrichment pathway analysis. This gave an overall statistical value for each individual pathway to aid in identifying which pathways were the most significantly enriched in AD based on both the DE-miRNA and DE-protein findings in the current review (Figure 5). Notably, the hippo-signaling pathway (*p* = 1.66 × 10^−9^) was the most significant by a considerable amount.

A final table of results was made with miRNA–protein inverse correlations (Table 3).

## 4. Discussion

In this extensive parallel systematic review, we identified 28 studies including a total of 113 DE-miRNAs (53 validated by qRT-PCR) and 26 studies including 196 DE-proteins in human AD brains compared to healthy age-matched controls. A total of 39 common KEGG pathways were identified between the two studies, 28 of which included 249 miRNA-protein inverse relationships, representing 249 potential targets for therapeutic intervention. Among all DE-miRNAs, meta-analysis revealed that miR-132-3p and miR-16 are consistently downregulated in late-stage AD across the literature.

MiR-132-3p is a neuron-specific miRNA, associated with hippocampal formation, synaptic morphogenesis, and promotes neuronal growth in response to neurotrophins. Therefore, its implications in cognitive function make it an ideal target for AD research. MiRNA profiling of miR-132 is highly consistent across the board—unaffected by sex, APO genotype, and brain regions studied (TC, HC, FC). Two main proposed mechanisms are involvement with inflammation and apoptotic pathways. Wong et al. show that a downregulation of miR-132-3p results in the over-expression of pro-apoptotic genes FOXO3a, EP300, and PTEN that are direct targets of miR-132. These three pathways converge with the nuclear translocation of FOXO3 which becomes transcriptionally active resulting in caspase 3-dependent cleavage and neuronal apoptosis. Wong et al. 2013 propose this is regulated by activity-dependent neurotrophins CREB and BDNF, which positively regulate miR-132-3p expression and are downregulated in AD in response to Aβ-dependent synaptotoxicity [58]. Alternatively, downregulation of miR-132 is correlated with an upregulation of inflammatory signals.

Transcriptomic profiling by Annese et al. [43] identified that miR-132-3p has a complementary ‘5 seed region to IL-6R which is involved in inflammatory signaling and could constitute a potential mechanism of neuroinflammation in AD. However, Lau et al. [52] do not report co-localization of miR-132-3 downregulation with lesion sites in patients with multiple sclerosis (MS), a chronic inflammatory disease often used to study inflammatory aspects of neurodegenerative diseases, suggesting that miR-132-3p is not associated with inflammation in the CNS in AD. KEGG pathway and gene target analysis in the DIANA and DAVID databases did not reveal any inverse relationships between downregulated miR-132-3p and upregulated proteins in the AD brain. Consequently, this study did not unveil any new applications for miR-132-3p involvement in AD pathology or highlight its potential as a therapeutic target.

MiR-16 was another amongst the most widely reported miRNAs found to be differentially expressed in the post-mortem brains of AD patients. Despite this observation, definitive mechanisms of miR-16 involvement in AD pathology are yet to be fully described. Moncini et al. [57] and Zhong et al. [33] contextualize their findings to possible miR-16 regulation of BACE1 gene encoding ß-secretase enzyme essential for the amyloidogenic cleavage of APP. Zhong et al. [33] confirm the inverse relationship between miR-16 and BACE1 expression through their PC12 cellular AD model which utilizes Western blots to show that miR-16 transfection of PC12 cells directly suppresses BACE1 expression and reduces cellular apoptosis. This confirms that miR-16 is neuroprotective and seems to be a promising target for further research into the development of AD therapies. Moncini et al. [57], however, report that overexpression of miR-16 does not reduce BACE1 expression as effectively as other miRNAs from the miR-15/107 family. Our co-expression network analysis revealed two inverse relationships between miR-16 and proteins in the AD brain. Crucially to this review, an inverse relationship was identified between downregulated miR-16 and upregulated CLOCK protein in the circadian rhythm and upregulated heat-shock protein A-4L (HSPA4L) in the Protein Processing in the Endoplasmic Reticulum Pathway.

Sleep and circadian rhythm disturbances are among the first symptoms of AD, often preceding motor and cognitive symptoms by years [94]. Patients experience increasingly fragmented night-time sleep coupled with hours of nocturnal activity and daytime sleepiness. Lastly, prolonged sleep and periods of unconsciousness are common days before death of AD patients. In addition, circadian rhythm is controlled in every cell by the peripheral oscillators CLOCK and brain and muscle ARNTL-like 1 (BMAL1) which form an autonomous, negative feedback transcriptional network [95,96]. CLOCK and BMAL1 are transcriptional activators expressed in the cytoplasm of all cells. Heterodimerization of CLOCK and BMAL1 results in their translocation to the nucleus where they bind to the E-box enhancer element in the promotor region of a plethora of genes including PERIOD (Per) and Cryptochrome (Cry) to induce their transcription. Per and Cry proteins accumulate in the cytoplasm during the day until they reach a critical concentration in the late afternoon whereby, they heterodimerize and translocate to the nucleus where they suppress the transcription of CLOCK and BMAL1. This cycle maintains the 24 h transcriptional rhythm of every cell. Previous studies also suggested an involvement of CLOCK/BMAL1 in AD [77,97,98].

Our co-expression analysis showed that CLOCK and BMAL1 are upregulated in the AD brain. Crucially, CLOCK has an inverse relationship with miR-16 which is consistently downregulated in the AD brain. We suggest that overexpression of CLOCK and BMAL1 disrupts the circadian rhythm generation by peripheral oscillators and induces prolonged periods of transcriptional day-time activity. As CLOCK and BMAL1 must heterodimerize to induce transcription, targeting CLOCK alone would be effective enough to alter transcriptional profiles of cells. Daily mid-afternoon administration of an miR-16 mimic with a short half-life would degrade CLOCK mRNA transcripts and reduce its cellular concentration switching cells to a night-time expression profile by evening. This could be considered as a potential therapeutic intervention. Interestingly, the CLOCK/BMAL1 dimer regulates transcription of the presenilin 2 gene (PSN2), regulating in APP cleavage [99]. Therefore, suppression of CLOCK/BMAL1 will reduce the rate of Aβ production and stabilize downstream effects such as regulation of intracellular calcium levels.

Additionally, our study revealed inverse relationships between overexpressed CSNK1e and miRNA in the circadian rhythm pathway. CSNKIE hyperphosphorylates the PER and CRY proteins targeting them for E3 ubiquitination by βTrCP and degradation by the 26S proteosome [100,101]. Excessive degradation of Cry and Per prevents their suppression of CLOCK and BMAL1 transcription, increasing the amount of CLOCK/BMAL1 bound to E-box promotor region and prolonging the daytime transcriptional profile of cells. Therefore, late afternoon administration of hsa-miR-329-3p or hsa-miR-495-3p miRNA mimics could suppress CSNK1e translation and indirectly reduce evening CLOCK and BMAL1 levels. Studies also show that CSNK1e regulates the timing and duration of sleep as overexpression of CSNK1e in Tau/Tau mice decreased the number of bouts of sleep and decreased duration of REM sleep [102] of hsa-miR-329-3p and hsa-miR-495-3p miRNA mimics could increase sleep duration and quality. In contrast to this, in vitro studies with CSNK1e specific inhibitor (PF-4800567) showed a minimal alteration of the circadian period, so other targets should be considered within this pathway [103].

Another important finding was the Hippo signaling pathway showing the most significant *p* value generated by the DIANA database *p* = 7.91 × 10^−8^, and it was one of the few pathways that received a *p* value and was ‘enriched’ in the DAVID database *p* = 0.021. These combined *p* values generate a value of *p* = 1.66 × 10^−9^ which is extremely statistically significant and shows that the Hippo pathway is highly likely to be affected by dysregulated miRNA and protein expression in the AD brain. The Hippo signaling pathway kinase cascade pathway responsible for the regulation of organ size and is evolutionarily conserved across a range of species. First identified in *Drosophila melanogaster* flies, the pathway is activated at high tissue densities to restrict organ size and prevent over-growth [104]. For this reason, the Hippo pathway also plays important roles in tissue homeostasis, tumor suppression, cellular proliferation, survival, and apoptosis. The pathway hinges around transcriptional co-activators yes-associated protein (YAP), and transcriptional coactivator with PDZ-binding motif (TAZ), or YAP/TAZ [105]. In Hippo’s inactive state, YAP/TAZ are bound to a family of transcriptional enhancer factors (TEF) in the cell nucleus to promote cell proliferation and growth [106]. When the hippo pathway is activated, kinases MST1 and MST2, along with their LATS1 and LATS2 cofactors, phosphorylate YAP/TAZ to promote their translocation to the cytoplasm, preventing their promotion of survival genes and inducing E3 ubiquitin-mediated degradation of the cell [107,108]. This results in apoptosis and restriction of organ growth.

Our miRNA and protein bioinformatic analysis also showed that casein kinase 1 isoform epsilon (CSNK1e) protein expression is increased in the brains of AD patients. CSNK1e is known to phosphorylate a range of targets including YAP/TAZ [109]. This prevents their nuclear localization and results in cellular apoptosis. CSNK1e’s interactions with YAP/TAZ is further suggested to disrupt the balance of ubiquitination and deubiquitylation of YAP/TAZ, inducing proteolysis and signaling the cell for degradation [110]. Pathogenic activation of the Hippo signaling pathway could be a mechanism for the decreased hippocampal, prefrontal cortex, and temporal cortex volume seen in AD patients [111]. Furthermore, YAP/TAZ are known to regulate vascular development and the maturation of the BBB in early development, a mechanism that is quiescent in adulthood [112]. Reactivation of the Hippo pathway could inappropriately restrict blood flow to the brain and BBB size, promoting ischemia and BBB degeneration. This would cause widespread neuronal atrophy and render the brain vulnerable to peripheral inflammatory signaling and Aβ, perpetuating AD pathology.

The DIANA database predicted that hsa-miR-320a, hsa-miR-329-3p, and hsa-miR-495-3p have complementary 5′ seed regions to CSNK1e mRNA and are downregulated in AD displaying inverse relationships with CSNK1e expression. Thus, we hypothesize that induction of the above miRNA mimics would suppress CSNK1e translation to reduce YAP/TAZ phosphorylation and pathogenic activation of the Hippo pathway. This could promote cell survival signaling and protect the brain from ischemia, inflammation, and excessive Aβ accumulation from the peripheral circulation [113]. This is a novel therapeutic target for AD. While it is known that the Hippo pathway is dysregulated in AD, current research groups are far from proposing a treatment. It should be recognized that over-suppression of the Hippo pathway has been identified in several cancer types [114]. Thus, it is essential to ensure treatments only rebalance YAP/TAZ signaling and do not induce excessive survival-signaling and the proliferation of cancers.

### Limitations

There are several limitations of a systematic review and the in silico nature of this study. For example, miRNA gene targets were predicted using the MicroTDS database that is only 60% accurate; therefore, not only are interactions between miRNAs and proteins themselves merely hypothesized, but their co-localization and unity within KEGG pathways in the AD brain is yet to be experimentally validated. Our inclusion/exclusion criteria for this review limited studies to the last 10 years, which means that we may have missed some earlier studies and hence giving an incomplete picture of miRNA–protein interactions. Although we have documented transcriptional and post-translational changes in this systematic review, further studies should assess these changes in a combined way so as to resolve differences between transcriptional levels and eventual post-translation levels of each gene/protein. Future studies should also aim to expand their inclusion criteria to all miRNA and proteomics studies applicable regardless of completion date. Furthermore, the inclusion of AD miRNA and protein profiling was limited to patients in Braak stage IV and above. However, miRNA expression is Braak-stage specific and miR-132-3p and miR-16 are upregulated in early-stage AD contrary to their downregulation in late-stage AD detailed in this review. This weakens our conclusions regarding the applicability of miR-132-3p and miR-16 as biomarkers in AD and would reduce their effectiveness as early interventional drug targets.

## 5. Conclusions

This review has highlighted several key areas in the Alzheimer’s field which could drastically improve the understanding of the disease pathology and potentially contribute towards resolving the major issues hindering the development of disease-modifying treatments. Here, key pathways where protein dysregulation contributes to AD pathology have been examined and within them several co-expressed miRNAs that may be responsible for this dysregulation have been identified. Whilst identifying the change of protein expression in AD is not novel, this research has linked these proteins to DE-miRNAs that have the potential to regulate the protein expression. Therefore, further research into the relationships between the DEmiRNAs and the proteins they regulate in AD may underpin novel explanations for the protein expression changes observed in pathology. Understanding this may provide novel therapeutic targets which have the potential to restore protein expression to normal physiological levels without complete inhibition of certain protein functions and signaling cascades causing adverse effects.

The use of miRNA-based therapies to regulate the circadian rhythm and Hippo signaling pathways is worth perusing and may prove beneficial pharmaceutical interventions beyond AD.

## Figures and Tables

**Table 1:** Inclusion and exclusion criteria utilized to direct the miRNA and protein in AD systematic literature search.

Inclusion	Exclusion
Alzheimer’s disease	Braak score < iv CERAD score ≥ 3 Other neurodegenerative diseases—Parkinson’s Disease, Lewy body pathology, Huntington’s Disease, Mild Cognitive Impairment, normal aging, inflammatory diseases (Multiple Sclerosis), PART
Post-mortem brain analysis	Plasma, serum, CSF, Saliva, cell-lines, transfected tissues, tissue biopsy
qRT-PCR or protein analyses	RNAseq, microarray analysis, in-situ hybridization
Qualitative and quantitative analysis	Study focusing on post-translational modifications, mutations, allelic variants, study including treatment or intervention
Human	Animals, cell lines
Male and female participants	None
Age-matched controls compared to AD	Single cohort studies, case studies, non-age-matched controls
Age ≥ 60	Age < 60
All patient ethnicities	No ethnicities were excluded
Primary research	Reviews, meta-analyses, bioinformatics studies using previously collected data, conference abstracts, clinical trials
Sample size *n* ≥ 3	Sample size *n* < 3
Published in peer-reviewed journals	Non-peer-reviewed
English language	Not written in English

**Table 2:** Characteristics of the studies. Data extraction contains: reference; patient information for participants in each study including number of participants (N), mean age, gender (M:F) for AD and control group; brain regions; analysis methods, and up- and downregulation of miRNAs (**A**) and proteins (**B**).

(A): Up and Downregulation of miRNAs						
Author	AD group (N; Mean Age; M:F)	Control group (N; Mean Age; M:F)	Brain Regions	Methods	miRNA Upregulated	miRNA Downregulated
Annese et al. 2018 [43]	14; 74; 8:5	14; 77; 8:5	HC; MTG; MFG	qRT-PCR	miR-10a-5p, miR-28-3p	miR-132-3p, miR-132-5p, miR-184, miR-212-3p, miR-212-5p, miR-34c-3p, miR-375, miR-539-5p
Cheng et al. 2020 [44]	8; 76; 3:8	8; 67; 4:5	FC; BDE	qRT-PCR	miR-17-5p, miR-18a-5p, miR-190a-5p, miR-219a-2-3p, miR-3157-5p, miR-374b-5p, miR-374c-3p, miR-548, miR-550a-3p, miR-550b-2-5p	miR-4284, miR-5001-3p, miR-132-5p
Chopra et al. 2020 [45]	29; 84; 11:18	25; 86; 9:16	TC; CB	qRT-PCR		miR-298
Culpan et al. 2011 [46]	12; 82; 5:7	6; 88; 5:1	FNC; TNC	qRT-PCR		miR-128a, miR-128b
Gong et al. 2017 [47]	40; -; -	35; -; -	FC	qRT-PCR		miR-15b
Herbert et al. 2013 [48]	8; 78; 5:3	8; 71; 5:3	STG; MTG	qRT-PCR		miR-132-3p, miR-100
Henriques et al. 2020 [49]	16; 81; 4:12	18; 78: 6:12	STG; MTG	qRT-PCR	miR-3651	miR-1202, miR-30e-3p, miR-365b-5p, miR-4286, miR-4443, miR-4449, miR-664-3p, miR-767-5p,
Kumar et al. 2018 [50]	27; 80; 14:13	15; 79; 8:7	FC	qRT-PCR	miR-455-3p	
Kumar et al. 2017 [51]	12; 80; 4:8	5; 73; 3:2	FC	qRT-PCR	miR-3613-3p, miR-455-3p, miR-4674, miR-6722	miR-122-5p
Lau et al. 2013 [52]	41; -; -	23; -; -	FC; HC	qRT-PCR	miR-142-3p, miR-200a-3p, miR-27a-3p, miR-92b-3p	miR-124-3p, miR-128, miR-129-2-3p, miR-129-5p, miR-132-3p miR-136-5p, miR-138-5p
Lei et al. 2015 [53]	31; 78; 18:13	29; 80; 16:13	FC	qRT-PCR		miR-29c
Li et al. 2019 [54]	30; 88; 18:12	30; 87; 20:10	FC	qRT-PCR		miR-219-5p
Liu et al. 2019 [55]	10; -; -	10; -; -	-	qRT-PCR		miR-132
Llorens et al. 2017 [56]	25; -; -	25; -; -	LC; EC; HC	qRT-PCR	miR-124-3p, miR-132-3p, miR-143-3p, miR-27a-3p	miR-124-3p
Long et al. 2019 [28]	15; 84; -	5; 84; -	FC	qRT-PCR		miR-346
Moncini et al. 2016 [57]	12; 78; 7:3	11; 82; 4:7	HC; TC	qRT-PCR		miR-103, miR-107, miR-15b, miR-16, miR-195
Muller et al. 2014 [58]	10; 78; 7:3	11; 83; 4:7	HC	qRT-PCR	miR-16 miR-146	miR-16 miR-146 miR-107 miR-128a
Pichker et al. 2017 [59]	39; 80; 15:24	25; 65; 15:10	TC; PFC	qRT-PCR		miR-132 miR-212-3p
Qian et al. 2019 [60]	12; 81; -	11; 82; -	HC	qRT-PCR		miR-338-5p
Santa-Maria et al. 2015 [61]	7; 93; 3:4	20; 89; 9:11	FC	qRT-PCR		miR-219-5p
Sarkar et al. 2016 [27]	13; 76; 6:7	10; 77; 5:5	TC; FC; CB	qRT-PCR	miR-146a	miR-132
Wang et al. 2018 [62]	12; 86; 3:9	12; 86; 1:11	TC; HC	qRT-PCR	miR-124	
Wong et al. 2013 [63]	16; 81; 6:10	16; 77; 10:6	TC	qRT-PCR		miR-132 miR-212
Yuan et al. 2020 [64]	10; 75; 6:4	10; 80; 6:4	-	qRT-PCR	miR-425-5p	
Zhang et al. 2016 [65]	7; 87; 3:4	7; 87; 1:16	HC	qRT-PCR	miR-603	
Zhao et al. 2016 [66]	12; 74; -	6; 72; -	TC; HC	qRT-PCR	miR-7 miR146a miR-155	
Zhao et al. 2013 [67]	3; 72; -	3; 72; -	HC	qRT-PCR	miR-34a miR-146a miR-125b miR-155	
Zhong et al. 2018 [33]	30; 87; -	20; 87; -	FC	qRT-PCR		miR-16
**(B): Up and downregulation of proteins**						
**Author**	**AD group** **(N; Mean Age; M:F)**	**Control group** **(N; Mean Age; M:F)**	**Brain Regions **	**Methods**	**Protein** **Upregulated**	**Protein** **Downregulated**
Beckelman et al. 2016 [68]	5; 82-98; 2:3	5; 78-97; 3:2	TC	WB, IHC		EEF1A1
Chiu et al. 2015 [69]	7; 82.9; 3:4	8; 61-91; 10:4	HP	IHC		ABCB1 (P-Glycoprotein)
Shepherd et al. 2020 [70]	17; 78; -	16; 74; -	TC	WB, ELISA	APP, MAPT	RAP
Chen et al. 2012 [71]	18; 74-89; -	13; 68-69; -	HP, FL, TL, CB	ELISA	NF-κb, BACE1	
Holler et al. 2014 [72]	52; 85.9; 19:33	19; 85.2; 5:14	HP	Immunoblot/IHC		BIN1
Walker et al. 2015 [73]	12; 78,9; 6:6	12; 84; 9:3	TC	WB	SOCS4, SOCS7	
Glennon et al. 2013 [74]	24; 69-96; 6:18	24; 76.4; 14:10	HP	Immunoblot		BIN1
Byman et al. 2018 [75]	12; 63-96; 3:9	8; 60-102; 5:3	HP, IP, IT, FC, SMTG	ELISA, IHC	AMY1A	
Huang et al. 2020 [76]	26; 88.6; 12:14	19; 90.3; 9:10	FC	WB, IP, IHC, IF	RBM15B	METTL3
Yoo et al. 2020 [77]	3; 72; 0:3	3; 65; 2:1	FC	IF	CLOCK, BMAL1	
Chen et al. 2012 [78]	12; 68-92; 8:4	12; 81-92; 9:3	FC, TC, PC, OC	Mass spectrometry	CLU	
Gu et al. 2020 [79]	10; 76.6; 6:4	9; 79.22; 4:6	FC	WB, IHC	CK1ε	TDP43
Xu et al. 2019 [80]	9; 60-80; 6:3	9; 61-78; 5:4	HP, EC, CG, SCx, MCx, CB	MS	AGT, AHNAK, ALAD, ANXA5, AQP4, ASAH1, BAG3, C3, CHGA, CLU, CP, DBI, DKK3, ESD, FGA, FGB, FGG, GJA1, H3F3A, HDGF, HIST1H1C, HIST1H1E, HP, HPX, HRSP12, HSPA1A, HSPB1, IGHA1, IGHG1, IGKC, ISYNA1, ITIH4, MAOB, MAP4, MARCKS, MECP2, NAMPT, NUCKS1, ORM1, PADI2, PAICS, PBXIP1, PCBD1, PLIN3, PNPO, PRDX1, PRDX6, S100A1, S100A11, S100A6, S100A9, SAA1, SELENBP1, SERPINA1, SERPINA3, SERPING1, SPR, STOM, TPD52L1	ACTN2, ADAP1, AP1G1, CADPS, CAP2, CIRBP, CORO1A, CORO2B, CRAT, DLAT, DLG4, DNAJC6, DNM3, DUSP3, EEF1B2, FARSB, GAS7, GLS, GRPEL1, HGS, HOMER1, HSPA4L, IARS2, IDH3G, IPO7, KIAA0513, KIF5C, LONP1, LRPPRC, LZTFL1, MAPRE3, NDUFA10, NECAB1, OAT, OGDH, OGDHL, OTUB1, OXCT1, PAFAH1B1, PDHX, PDIA3, PHYHIPL, PPME1, PPP2R1A, PTPA, PREP, PRKRA, RAP1GDS1, RGS7, RPH3A, SARS2, SCAI, SDR39U1, SGTB, SH3GL1, SLIRP, SMS, STXBP1, STXBP3, SUCLA2, SUCLG1, TIMM44, TLN2, TRAP1, VPS35, YARS, YWHAG, YWHAH, YWHAQ
Batkulwar et al. 2018 [81]	3; 84.3; -	3; 89.3; -	FC	MS	CML, Cathepsin B, AEP, RAGE, TAU	
Ilic et al. 2019 [82]	6; 77.8; 2:4	6; 75.5; 2:4	-	IHC	NPTN	
Lue et al. 2015 [83]	11; 82.46; 9:13	11; 85.4; 7:4	FC	Immunoblot	TREM2, DAP12, IBA1, CASP3	SNAP25, PSD95
Bekris et al. 2010 [84]	8; 60-93; 5:3	8; 79-94; 4:4	HP	WB		APOE
Causevic et al. 2010 [85]	4; 82-97; -	4; 81-86; -	HP	WB		IDE
Campanari et al. 2016 [86]	19; 75-85; 8:11	22; 65-73; 12:10	FC	WB	ACHE	
Bartolotti et al. 2016 [87]	21; 93.1; 0:21	20; 93.49; 0:20	CB, FC	WB		CREB, CBP, EP300
Jin et al. 2013 [88]	7; 86.29; 1:6	7; 86.6; 2:5	FC	WB		GLUT3
Gu et al. 2020 [89]	12; 75-98; 3:9	12; 61-100; 3:9	TC	WB, & IHC		YWHAG, YWHAH (14-3-3 Proteins)
Ginsberg et al. 2010 [90]	38; 84.6; 14:24	27; 80.8; 5:12	PFC	Quantitative immunoblot	RAB5A, RAB7A	
Wang et al. 2010 [91]	10; 87.3; 3:7	10; 80.5; 7:3	HP, EC, CG, SCx, MCx, CB	WB		NEP, IDE
Sengupta et al. 2018 [92]	4; 75-83; 3:1	4; 70-79; 2:2	HP, BF, FC, CB, STR	WB, IF	MSI1, MSI2	
Liao, et al. 2016 [93]	10; 81.8; 4:6	7; 83.6; 3:4	MTG	WB, IHC, ELISA	NF-κB, MCP-1, MIP1α	

Brain region: HC = hippocampus, TC = temporal cortex, MFG = medial temporal gyrus, MFG = medial frontal gyrus, FC = frontal cortex, CB = cerebellum, FNC = frontal neo cortex, STG = superior temporal gyrus, BDE = brain-derived exosome.

**Table 3:** MiRNA–protein inverse relation of the 28 common pathways. ↓ = down-regulation; ↑ = up-regulation; “–” = Not Determined.

Common Pathways	miRNA *p* Value	Protein *p* Value	miRNA (−log (p Value)	miRNA-Protein Inverse Relation
Hippo signaling pathway	7.91 × 10^−8^	0.021	7.1	↓ miR-320a [43,44], miR-329-3p [52], miR-495-3p [52] ↑ CSNK1E [79]
				↑ miR-3613-3p [51], miR-200a-3p [52], miR-199a-3p [44,52], miR-199b-3p [52], miR-23a-3p [44,52], miR-425-5p [52,64], miR-34c-3p [43,44,56] ↓ YWHAG [80,89]
				↑ miR-3613-3p [51] ↓ YWHAH [80,89]
				↑ miR-27a-3p [52,56], miR-455-3p [50,51] ↓ YWHAQ [80]
				↑ miR-150-5p [52], ↓ PPP2R1A [80]
Pathways in cancer	9.57 × 10^−6^	-	5	↑ miR-3613-3p [51], miR-23a-3p [44,52], miR-550a-3p [34] ↓ CREBBP [87]
				↑ miR-603 [65], miR-3613-3p [51] ↓ EP300 [92]
Adherends junction	2.33 × 10^−5^	-	4.6	↑ miR-23a-3p [44,52] ↓ ACTN2 [80]
				↑ miR-23a-3p [44,52], miR-3613-3p [51], miR-550a-3p [34] ↓ CREBBP [87]
				↑ miR-603 [65], miR-3613-3p [51] ↓ EP300 [87]
Wnt signaling pathway	0.001	-	3.1	↓ miR-495-3p [52], miR-329-3p [53], miR-320a [43,44], ↑ CSNK1E [79]
				↑ miR-603 [65], miR-3613-3p [51] ↓ EP300 [87]
				↑ miR-3613-3p [51], miR-23a-3p [44,52], miR-550a-3p [34] ↓ CREBBP [87]
PI3K-Akt signaling pathway	0.001	-	3	↑ miR-27a-3p [52,56], miR-10a-5p [43], miR-374b-5p [34], miR-155-5p [66,67], miR-200a-3p [52], miR-3613-3p [51], miR-362-3p [52], miR-425-5p [52,64] ↓ CREBBP [87]
				↑ miR-150-5p [52] ↓ PPP2R1A [80]
				↑ miR-199a-3p [44,52], miR-199b-3p [52], miR-200a-3p [52], miR-3613-3p [51], miR-23a-3p [44,52], miR-425-5p [52,64], miR-34c-3p [43,44,56] ↓ YWHAG [80,89]
				↑ miR-3613-3p [51] ↓ YWHAH [80,89]
				↑ miR-27a-3p [52,56], miR-455-3p [50,51] ↓ YWHAQ [70]
GABAergic	0.001	-	3	↑ miR-200a-3p [52], miR-9-5p [27], miR-125b-5p [67] ↓ GLS [80]
Estrogen signaling pathway	0.002	-	2.7	↑ miR-155-5p [66,67], miR-27a-3p [52,56], miR-3613-3p [51], miR-374b-5p [34], miR-10a-5p [43], miR-200a-3p [52], miR-425-5p [52,64], miR-362-3p [52] ↓ CREB1 [87]
Thyroid hormone signaling pathway	0.002	-	2.7	↑ miR-3613-3p [51], miR-23a-3p [44,52], miR-550a-3p [34] ↓ CREBBP [87]
				↑ miR-155-5p [62,66,67], miR-27a-3p [52,56], miR-3613-3p [51], miR-374b-5p [34], miR-10a-5p [43], miR-200a-3p [52], miR-425-5p [52,64], miR-362-3p [52] ↓ CREB1 [87]
Prolactin signaling pathway	0.002	-	2.6	↓ miR-487a-3p [52], miR-136-5p [52], miR-543 [52], miR-889-3p [43] ↑SOCS4 [73]
Protein processing in endoplasmic reticulum	0.002	-	2.6	↓ miR-219a-2-3p [34,52], miR-107 [56,57], miR-103a-3p [57], miR-30e-3p [49], miR-30a-3p [43], miR-195-5p [52,57], miR-16-5p [33,56,57], miR-15b-5p [47,57], miR-889-3p [43], miR-539-5p [43], miR-410-3p [52], miR-129-5p [52], miR-543 [52], miR-375 [43], miR-17-5p [34], miR-495-3p [52], miR-338-5p [60], miR-320a [43,44] ↑ HSPA4L [80]
Endocytosis	0.004	0.002	2.4	↓ miR-298 [45], miR-539-5p [43], miR-18a-5p [34], miR-582-5p [43] ↑RAB5A [90]
				↑ miR-603 [65], miR-23a-3p [44,52], miR-3613-3p [51] ↓ DNAJC6 [80]
				↑ miR-3613-3p [51], miR-23a-3p [44,52], miR-548 [34], miR-603 [65], miR-362-3p [52], miR-27a-3p [52,56], miR-146a-3p [27,56,67] ↓ DNM3 [80]
AMPK signaling pathway				↑ miR-142-3p [52] ↓ HGS [80]
AMPK signaling pathway	0.005	-	2.3	↑ miR-425-5p [52,64], miR-155-5p [66,67], miR-27a-3p [52,56], miR-10a-5p [43], miR-362-3p [52], miR-374b-5p [34], miR-3613-3p [51], miR-200a-3p [52] ↓ CREB1 [87]
AMPK signaling pathway FoxO signaling pathway				↑ miR-150-5p [52] ↓ PPP2R1A [80]
AMPK signaling pathway FoxO signaling pathway	0.006	-	2.2	↓ miR-329-3p [52], miR-495-3p [52], miR-320a [43,44] ↑ CSNK1E [79]
AMPK signaling pathway FoxO signaling pathway				↑ miR-550a-3p [34], miR-3613-3p [51], miR-23a-3p [44,52] ↓ CREBBP [87]
AMPK signaling pathway FoxO signaling pathway				↑ miR-603 [65], miR-3613-3p [51] ↓ EP300 [87]
AMPK signaling pathway FoxO signaling pathway Adrenergic signaling in cardiomyocytes				↑ miR-374b-5p [34], miR-3613-3p [51], miR-34c-3p [43,44,56] ↓ HOMER1 [80]
AMPK signaling pathway FoxO signaling pathway	0.001	-	2.1	↑ miR-10a-5p [43], miR-425-5p [52,64], miR-374b-5p [34], miR-362-3p [52], miR-200a-3p [52], miR-155-5p [66,67], miR-27a-3p [52,56], miR-3613-3p [51] ↓ CREB1 [87]
Arrhythmogenic right ventricular cardiomyopathy (ARVC)				↑ miR-150-5p [52] ↓ PPP2R1A [80]
Arrhythmogenic right ventricular cardiomyopathy (ARVC) Transcriptional mis-regulation in cancer	0.008	-	2.1	↓ miR-320a [43,44], miR-543 [52], miR-582-5p [43], miR-889-3p [43], miR-410-3p [52], miR-539-5p [43], miR-30a-3p [43], miR-30e-3p [49], miR-329-3p [52], miR-298 [45], miR-338-5p [60] ↑ CREB1 [87]
Arrhythmogenic right ventricular cardiomyopathy (ARVC) Transcriptional mis-regulation in cancer	0.009	-	2	↓ miR-15b-5p [47,57] ↑ H3F3A [80]
TGF-beta signaling pathway	0.011	-	1.9	↑ miR-550a-3p [34], miR-3613-3p [51], miR-23a-3p [44,52] ↓ CREBBP [87]
				↑ miR-603 [65], miR-3613-3p [51] ↓ EP300 [87]
				↑ miR-150-5p [52] ↓ PPP2R1A [80]
Prostate cancer	0.011	-	1.9	↑ miR-425-5p [52,64], miR-10a-5p [43], miR-200a-3p [52], miR-374b-5p [34], miR-362-3p [52], miR-27a-3p [52,56], miR-3613-3p [51], miR-155-5p [66,67] ↓ CREB1 [87]
				↑ miR-550a-3p [34], miR-23a-3p [44,52], miR-3613-3p [51] ↓ CREBBP [87]
				↑ miR-603 [60], miR-3613-3p [46] ↓ EP300 [82]
cAMP signaling pathway	0.013	-	1.9	↑ miR-155-5p [61,62], miR-10a-5p [38], miR-200a-3p [47], miR-374b-5p [29], miR-27a-3p [47,51], miR-425-5p [47,50], miR-362-3p [47], miR-3613-3p [46] ↓ CREB1 [87]
				↑ miR-550a-3p [34], miR-23a-3p [44,52], miR-3613-3p [51] ↓ CREBBP [87]
				↑ miR-603 [65], miR-3613-3p [51] ↓ EP300 [87]
Cholinergic synapse	0.015	-	1.8	↑ miR-155-5p [66,67], miR-10a-5p [43], miR-200a-3p [52], miR-374b-5p [34], miR-27a-3p [52,56], miR-425-5p [52,55], miR-362-3p [52], miR-3613-3p [51] ↓ CREB1 [87]
Amoebiasis	0.020	0.004	1.7	↓ miR-18a-5p [34], miR-582-5p [43], miR-539-5p [43], miR-298 [45] ↑ RAB5A [90]
				↑ miR-23a-3p [44,52] ↓ ACTN2 [80]
Gap junction	0.021	-	1.7	↓ miR-539-5p [43], miR-664a-3p [49], miR-582-5p [43], miR-495-3p [52] ↑ GJA1 [80]
mRNA surveillance pathway	0.024	-	1.6	↓ miR-410-3p [52], miR-129-5p [52], miR-582-5p [43], miR-769-5p [52], miR-889-3p [43], miR-128-3p [52], miR-320a [43,44], miR-495-3p [52] ↑ MSI2 [92]
				↑ miR-150-5p [52] ↓ PPP2R1A [80]
Circadian rhythm	0.025	0.001	1.6	↓ miR-136-5p [52] ↑ ARNTL [77]
				↓ miR-15b-5p [47,57], miR-195-5p [52,59], miR-16-5p [33,56,57], miR-889-3p [43], miR-543 [52], miR-338-5p [60], miR-29c-3p [53], miR-129-5p [52], miR-495-3p [52], miR-107 [56,57], miR-103a-3p [57] ↑ CLOCK [77]
				↓ miR-329-3p [52], miR-495-5p [52] ↑ CSNK1E [79]
				↑ miR-27a-3p [52,56], miR-10a-5p [43], miR-374b-5p [34], miR-155-5p [66,67], miR-200a-3p [52], miR-3613-3p [51], miR-362-3p [52], miR-425-5p [52,64] ↓ CREB1 [87]
Insulin signaling pathway	0.027	-	1.6	↓ miR-487a-3p [52], miR-136-5p [52], miR-543 [52], miR-889-3p [43] ↑ SOCS4 [73]
Bacterial invasion of epithelial cells	0.352	-	1.5	↑ miR-603 [65], miR-23a-3p [44,52], miR-548 [34], miR-362-3p [52], miR-3613-3p [51], miR-27a-3p [52,56], miR-146a-3p [27,56,67] ↓ DNM3 [80]
cGMP-PKG signaling pathway	0.035	-	1.4	↑ miR-155-5p [66,67], miR-10a-5p [43], miR-200a-3p [52], miR-374b-5p [34], miR-27a-3p [52,56], miR-425-5p [52,64], miR-362-3p [52], miR-3613-3p [51], ↓ CREB1 [87]

## Data Availability

All data generated as part of this study are included in the article.

## Supplementary Materials

The following are available online at https://www.mdpi.com/article/10.3390/cells10123479/s1, Figure S1: miR-146a meta-analysis; Table S1: Full list of DE-miRNAs in the 28 selected studies. Table S2A,B: AXIS quality appraisal tool results for miRNA and protein studies; Table S3A,B: Risk of bias in miRNA and protein studies; Table S4A–D: Study characteristics of miRNAs selected for meta-analysis.

## References

[1] Alzheimer’s Association (2020). Alzheimer’s disease facts and figures. Alzheimers Dement..

[2] Hebert L.E., Weuve J., Scherr P.A., Evans D.A. (2013). Alzheimer disease in the United States (2010–2050) estimated using the 2010 census. Neurology.

[3] Virani S.S., Alonso A., Benjamin E.J., Bittencourt M.S., Callaway C.W., Carson A.P., Chamberlain A.M., Chang A.R., Cheng S., Delling F.N. (2020). Heart disease and stroke statistics—2020 update: A report from the american heart association. Circulation.

[4] Jennings L.A., Hollands S., Keeler E., Wenger N.S., Reuben D.B. (2020). The effects of dementia care co-management on acute care, hospice, and long-term care utilization. J. Am. Geriatr. Soc..

[5] Yu H., Wang X., He R., Liang R., Zhou L. (2015). Measuring the caregiver burden of caring for community-residing people with Alzheimer’s disease. PLoS ONE.

[6] GBD 2016 Dementia Collaborators (2019). Global, regional, and national burden of Alzheimer’s disease and other dementias, 1990–2016: A systematic analysis for the global burden of disease study 2016. Lancet Neurol..

[7] Viña J., Lloret A. (2010). Why women have more Alzheimer’s disease than men: Gender and mitochondrial toxicity of amyloid-beta peptide. J. Alzheimers Dis..

[8] Farrer L.A., Cupples L.A., Haines J.L., Hyman B., Kukull W.A., Mayeux R., Myers R.H., Pericak-Vance M.A., Risch N., van Duijn C.M. (1997). Effects of age, sex, and ethnicity on the association between apolipoprotein E genotype and Alzheimer disease. A meta-analysis. APOE and Alzheimer disease meta analysis consortium. JAMA.

[9] Evans D.A., Bennett D.A., Wilson R.S., Bienias J.L., Morris M.C., Scherr P.A., Hebert L.E., Aggarwal N., Beckett L.A., Joglekar R. (2003). Incidence of Alzheimer disease in a biracial urban community: Relation to apolipoprotein E allele status. Arch. Neurol..

[10] Matthews K.A., Xu W., Gaglioti A.H., Holt J.B., Croft J.B., Mack D., McGuire L.C. (2019). Racial and ethnic estimates of Alzheimer’s disease and related dementias in the United States (2015–2060) in adults aged ≥65 years. Alzheimers Dement..

[11] Breteler M.M., Claus J.J., Grobbee D.E., Hofman A. (1994). Cardiovascular disease and distribution of cognitive function in elderly people: The rotterdam study. BMJ.

[12] Wilson R.S., Krueger K.R., Arnold S.E., Schneider J.A., Kelly J.F., Barnes L.L., Tang Y., Bennett D.A. (2007). Loneliness and risk of Alzheimer disease. Arch. Gen. Psychiatry.

[13] Gottesman R.F., Albert M.S., Alonso A., Coker L.H., Coresh J., Davis S.M., Deal J.A., McKhann G.M., Mosley T.H., Sharrett A.R. (2017). Associations between midlife vascular risk factors and 25-year incident dementia in the atherosclerosis risk in communities (ARIC) cohort. JAMA Neurol..

[14] Zhao M., Veeranki S.P., Magnussen C.G., Xi B. (2020). Recommended physical activity and all cause and cause specific mortality in US adults: Prospective cohort study. BMJ.

[15] Mann D.M., Yates P.O., Marcyniuk B. (1985). Correlation between senile plaque and neurofibrillary tangle counts in cerebral cortex and neuronal counts in cortex and subcortical structures in Alzheimer’s disease. Neurosci. Lett..

[16] Hardy J., Allsop D. (1991). Amyloid deposition as the central event in the aetiology of Alzheimer’s disease. Trends Pharmacol. Sci..

[17] Rubin R. (2021). Recently approved alzheimer drug raises questions that might never be answered. JAMA.

[18] Makin S. (2018). The amyloid hypothesis on trial. Nature.

[19] Serrano-Pozo A., Das S., Hyman B.T. (2021). APOE and Alzheimer’s disease: Advances in genetics, pathophysiology, and therapeutic approaches. Lancet Neurol..

[20] Mirzaei S., Zarrabi A., Hashemi F., Zabolian A., Saleki H., Ranjbar A., Saleh S.H.S., Bagherian M., Sharifzadeh S.O., Hushmandi K. (2021). Regulation of nuclear Factor-KappaB (NF-κB) signaling pathway by non-coding RNAs in cancer: Inhibiting or promoting carcinogenesis?. Cancer Lett..

[21] Ashrafizadeh M., Zarrabi A., Hushmandi K., Hashemi F., Moghadam E.R., Owrang M., Hashemi F., Makvandi P., Goharrizi M.A.S.B., Najafi M. (2021). Lung cancer cells and their sensitivity/resistance to cisplatin chemotherapy: Role of microRNAs and upstream mediators. Cell. Signal..

[22] Mirzaei S., Zarrabi A., Asnaf S.E., Hashemi F., Zabolian A., Hushmandi K., Raei M., Goharrizi M.A.S.B., Makvandi P., Samarghandian S. (2021). The role of microRNA-338-3p in cancer: Growth, invasion, chemoresistance, and mediators. Life Sci..

[23] Catalanotto C., Cogoni C., Zardo G. (2016). MicroRNA in control of gene expression: An overview of nuclear functions. Int. J. Mol. Sci..

[24] Cao D.D., Li L., Chan W.Y. (2016). MicroRNAs: Key regulators in the central nervous system and their implication in neurological diseases. Int. J. Mol. Sci..

[25] Im H.I., Kenny P.J. (2012). MicroRNAs in neuronal function and dysfunction. Trends Neurosci..

[26] Hébert S.S., Sergeant N., Buée L. (2012). MicroRNAs and the regulation of tau metabolism. Int. J. Alzheimers Dis..

[27] Sarkar S., Jun S., Rellick S., Quintana D.D., Cavendish J.Z., Simpkins J.W. (2016). Expression of microRNA-34a in Alzheimer’s disease brain targets genes linked to synaptic plasticity, energy metabolism, and resting state network activity. Brain Res..

[28] Long J.M., Maloney B., Rogers J.T., Lahiri D.K. (2019). Novel upregulation of amyloid-β precursor protein (APP) by microRNA-346 via targeting of APP mRNA 5′-untranslated region: Implications in Alzheimer’s disease. Mol. Psychiatry.

[29] Han C., Guo L., Yang Y., Guan Q., Shen H., Sheng Y., Jiao Q. (2020). Mechanism of microRNA-22 in regulating neuroinflammation in Alzheimer’s disease. Brain Behav..

[30] Nelson P.T., Wang W.X. (2010). MiR-107 is reduced in Alzheimer’s disease brain neocortex: Validation study. J. Alzheimers Dis..

[31] Fang M., Wang J., Zhang X., Geng Y., Hu Z., Rudd J.A., Ling S., Chen W., Han S. (2012). The miR-124 regulates the expression of BACE1/β-secretase correlated with cell death in Alzheimer’s disease. Toxicol. Lett..

[32] El Fatimy R., Li S., Chen Z., Mushannen T., Gongala S., Wei Z., Balu D.T., Rabinovsky R., Cantlon A., Elkhal A. (2018). MicroRNA-132 provides neuroprotection for tauopathies via multiple signaling pathways. Acta Neuropathol..

[33] Zhong Z., Yuan K., Tong X., Hu J., Song Z., Zhang G., Fang X., Zhang W. (2018). MiR-16 attenuates β-amyloid-induced neurotoxicity through targeting β-site amyloid precursor protein-cleaving enzyme 1 in an Alzheimer’s disease cell model. Neuroreport.

[34] Hanna J., Hossain G.S., Kocerha J. (2019). The Potential for microRNA Therapeutics and Clinical Research. Front Genet..

[35] Ghaffari M., Sanadgol N., Abdollahi M.A. (2020). Systematic review of current progresses in the nucleic acid-based therapies for neurodegeneration with implications for Alzheimer’s disease. Mini. Rev. Med. Chem..

[36] Nazem A., Mansoori G.A. (2011). Nanotechnology for Alzheimer’s disease detection and treatment. Insci. J..

[37] Lanford R.E., Hildebrandt-Eriksen E.S., Petri A., Persson R., Lindow M., Munk M.E., Kauppinen S., Ørum H. (2010). Therapeutic silencing of microRNA-122 in primates with chronic hepatitis C virus infection. Science.

[38] Liberati A., Altman D.G., Tetzlaff J., Mulrow C., Gøtzsche P.C., Ioannidis J.P., Clarke M., Devereaux P.J., Kleijnen J., Moher D. (2009). The PRISMA statement for reporting systematic reviews and meta-analyses of studies that evaluate healthcare interventions: Explanation and elaboration. BMJ.

[39] Downes M.J., Brennan M.L., Williams H.C., Dean R.S. (2016). Development of a critical appraisal tool to assess the quality of cross-sectional studies (AXIS). BMJ Open.

[40] Higgins J.P., Altman D.G., Gøtzsche P.C., Jüni P., Moher D., Oxman A.D., Savovic J., Schulz K.F., Weeks L., Sterne J.A. (2011). The cochrane collaboration’s tool for assessing risk of bias in randomised trials. BMJ.

[41] Vlachos I.S., Kostoulas N., Vergoulis T., Georgakilas G., Reczko M., Maragkakis M., Paraskevopoulou M.D., Prionidis K., Dalamagas T., Hatzigeorgiou A.G. (2012). DIANA miRPath v.2.0: Investigating the combinatorial effect of microRNAs in pathways. Nucleic Acids Res..

[42] Paraskevopoulou M.D., Georgakilas G., Kostoulas N., Vlachos I.S., Vergoulis T., Reczko M., Filippidis C., Dalamagas T., Hatzigeorgiou A.G. (2013). DIANA-microT web server v5.0: Service integration into miRNA functional analysis workflows. Nucleic Acids Res..

[43] Annese A., Manzari C., Lionetti C., Picardi E., Horner D.S., Chiara M., Caratozzolo M.F., Tullo A., Fosso B., Pesole G. (2018). Whole transcriptome profiling of late-onset Alzheimer’s disease patients provides insights into the molecular changes involved in the disease. Sci. Rep..

[44] Cheng L., Vella L.J., Barnham K.J., McLean C., Masters C.L., Hill A.F. (2020). Small RNA fingerprinting of Alzheimer’s disease frontal cortex extracellular vesicles and their comparison with peripheral extracellular vesicles. J. Extracell. Vesicles.

[45] Chopra N., Wang R., Maloney B., Nho K., Beck J.S., Pourshafie N., Niculescu A., Saykin A.J., Rinaldi C., Counts S.E. (2020). MicroRNA-298 reduces levels of human amyloid-β precursor protein (APP), β-site APP-converting enzyme 1 (BACE1) and specific tau protein moieties. Mol. Psychiatry.

[46] Culpan D., Kehoem P.G., Love S. (2011). Tumour necrosis factor-α (TNF-α) and miRNA expression in frontal and temporal neocortex in Alzheimer’s disease and the effect of TNF-α on miRNA expression in vitro. Int. J. Mol. Epidemiol. Genet..

[47] Gong G., An F., Wang Y., Bian M., Yu L.J., Wei C. (2017). miR-15b represses BACE1 expression in sporadic Alzheimer’s disease. Oncotarget.

[48] Hébert S.S., Wang W.X., Zhu Q., Nelson P.T. (2013). A study of small RNAs from cerebral neocortex of pathology-verified Alzheimer’s disease, dementia with lewy bodies, hippocampal sclerosis, frontotemporal lobar dementia, and non-demented human controls. J. Alzheimers Dis..

[49] Henriques A.D., Machado-Silva W., Leite R., Suemoto C.K., Leite K., Srougi M., Pereira A.C., Jacob-Filho W., Nóbrega O.T., Brazilian Aging Brain Study Group (2020). Genome-wide profiling and predicted significance of post-mortem brain microRNA in Alzheimer’s disease. Mech. Ageing Dev..

[50] Kumar S., Reddy P.H. (2018). MicroRNA-455-3p as a Potential Biomarker for Alzheimer’s disease: An update. Front. Aging Neurosci..

[51] Kumar S., Vijayan M., Reddy P.H. (2017). MicroRNA-455-3p as a potential peripheral biomarker for Alzheimer’s disease. Hum. Mol. Genet..

[52] Lau P., Bossers K., Janky R., Salta E., Frigerio C.S., Barbash S., Rothman R., Sierksma A.S., Thathiah A., Greenberg D. (2013). Alteration of the microRNA network during the progression of Alzheimer’s disease. EMBO Mol. Med..

[53] Lei X., Lei L., Zhang Z., Zhang Z., Cheng Y. (2015). Downregulated miR-29c correlates with increased BACE1 expression in sporadic Alzheimer’s disease. Int. J. Clin. Exp. Pathol..

[54] Li J., Chen W., Yi Y., Tong Q. (2019). miR-219-5p inhibits tau phosphorylation by targeting TTBK1 and GSK-3β in Alzheimer’s disease. J. Cell Biochem..

[55] Liu D.Y., Zhang L. (2019). MicroRNA-132 promotes neurons cell apoptosis and activates Tau phosphorylation by targeting GTDC-1 in Alzheimer’s disease. Eur. Rev. Med. Pharmacol. Sci..

[56] Llorens F., Thüne K., Andrés-Benito P., Tahir W., Ansoleaga B., Hernández-Ortega K., Martí E., Zerr I., Ferrer I. (2017). MicroRNA expression in the locus coeruleus, entorhinal cortex, and hippocampus at early and middle stages of braak neurofibrillary tangle pathology. J. Mol. Neurosci..

[57] Moncini S., Lunghi M., Valmadre A., Grasso M., Del Vescovo V., Riva P., Denti M.A., Venturin M. (2017). The miR-15/107 Family of microRNA genes regulates CDK5R1/p35 with Implications for Alzheimer’s disease pathogenesis. Mol. Neurobiol..

[58] Müller M., Kuiperij H.B., Claassen J.A., Küsters B., Verbeek M.M. (2014). MicroRNAs in Alzheimer’s disease: Differential expression in hippocampus and cell-free cerebrospinal fluid. Neurobiol. Aging.

[59] Pichler S., Gu W., Hartl D., Gasparoni G., Leidinger P., Keller A., Meese E., Mayhaus M., Hampel H., Riemenschneider M. (2017). The miRNome of Alzheimer’s disease: Consistent downregulation of the miR-132/212 cluster. Neurobiol. Aging.

[60] Qian Q., Zhang J., He F.P., Bao W.X., Zheng T.T., Zhou D.M., Pan H.Y., Zhang H., Zhang X.Q., He X. (2019). Down-regulated expression of microRNA-338-5p contributes to neuropathology in Alzheimer’s disease. FASEB J..

[61] Santa-Maria I., Alaniz M.E., Renwick N., Cela C., Fulga T.A., Van Vactor D., Tuschl T., Clark L.N., Shelanski M.L., McCabe B.D. (2015). Dysregulation of microRNA-219 promotes neurodegeneration through post-transcriptional regulation of tau. J. Clin. Investig..

[62] Wang X., Liu D., Huang H.Z., Wang Z.H., Hou T.Y., Yang X., Pang P., Wei N., Zhou Y.F., Dupras M.J. (2018). A novel MicroRNA-124/PTPN1 signal pathway mediates synaptic and memory deficits in Alzheimer’s disease. Biol. Psychiatry.

[63] Wong H.K., Veremeyko T., Patel N., Lemere C.A., Walsh D.M., Esau C., Vanderburg C., Krichevsky A.M. (2013). De-repression of FOXO3a death axis by microRNA-132 and -212 causes neuronal apoptosis in Alzheimer’s disease. Hum. Mol. Genet..

[64] Yuan J., Wu Y., Li L., Liu C. (2020). MicroRNA-425-5p promotes tau phosphorylation and cell apoptosis in Alzheimer’s disease by targeting heat shock protein B8. J. Neural. Transm..

[65] Zhang C., Lu J., Liu B., Cui Q., Wang Y. (2016). Primate-specific miR-603 is implicated in the risk and pathogenesis of Alzheimer’s disease. Aging.

[66] Zhao Y., Alexandrov P.N., Jaber V., Lukiw W.J. (2016). Deficiency in the ubiquitin conjugating enzyme UBE2A in Alzheimer’s Disease (AD) is linked to deficits in a natural circular miRNA-7 sponge (circRNA; ciRS-7). Genes.

[67] Zhao Y., Bhattacharjee S., Jones B.M., Dua P., Alexandrov P.N., Hill J.M., Lukiw W.J. (2013). Regulation of TREM2 expression by an NF-κB-sensitive miRNA-34a. Neuroreport.

[68] Beckelman B.C., Zhou X., Keene C.D., Ma T. (2016). Impaired eukaryotic elongation factor 1A expression in Alzheimer’s disease. Neurodegener. Dis..

[69] Chiu C., Miller M.C., Monahan R., Osgood D.P., Stopa E.G., Silverberg G.D. (2015). P-glycoprotein expression and amyloid accumulation in human aging and Alzheimer’s disease: Preliminary observations. Neurobiol. Aging.

[70] Shepherd C.E., Affleck A.J., Bahar A.Y., Carew-Jones F., Gregory G., Small D.H., Halliday G.M. (2020). Alzheimer’s amyloid-β and tau protein accumulation is associated with decreased expression of the LDL receptor-associated protein in human brain tissue. Brain Behav..

[71] Chen C.H., Zhou W., Liu S., Deng Y., Cai F., Tone M., Tone Y., Tong Y., Song W. (2012). Increased NF-κB signalling up-regulates BACE1 expression and its therapeutic potential in Alzheimer’s disease. Int. J. Neuropsychopharmacol..

[72] Holler C.J., Davis P.R., Beckett T.L., Platt T.L., Webb R.L., Head E., Murphy M.P. (2014). Bridging integrator 1 (BIN1) protein expression increases in the Alzheimer’s disease brain and correlates with neurofibrillary tangle pathology. J. Alzheimers Dis..

[73] Walker D.G., Whetzel A.M., Lue L.F. (2015). Expression of suppressor of cytokine signaling genes in human elderly and Alzheimer’s disease brains and human microglia. Neuroscience.

[74] Glennon E.B., Whitehouse I.J., Miners J.S., Kehoe P.G., Love S., Kellett K.A., Hooper N.M. (2013). BIN1 is decreased in sporadic but not familial Alzheimer’s disease or in aging. PLoS ONE.

[75] Byman E., Schultz N., Fex M., Wennström M., Netherlands Brain Bank (2018). Brain alpha-amylase: A novel energy regulator important in Alzheimer disease?. Brain Pathol..

[76] Huang H., Camats-Perna J., Medeiros R., Anggono V., Widagdo J. (2020). Altered Expression of the m6A Methyltransferase METTL3 in Alzheimer’s disease. eNeuro.

[77] Yoo I.D., Park M.W., Cha H.W., Yoon S., Boonpraman N., Yi S.S., Moon J.S. (2020). Elevated CLOCK and BMAL1 contribute to the impairment of aerobic glycolysis from astrocytes in Alzheimer’s disease. Int. J. Mol. Sci..

[78] Chen J., Wang M., Turko I.V. (2012). Mass spectrometry quantification of clusterin in the human brain. Mol. Neurodegener..

[79] Gu J., Hu W., Tan X., Qu S., Chu D., Gong C.X., Iqbal K., Liu F. (2020). Elevation of casein kinase 1ε associated with TDP-43 and tau pathologies in Alzheimer’s disease. Brain Pathol..

[80] Xu J., Patassini S., Rustogi N., Riba-Garcia I., Hale B.D., Phillips A.M., Waldvogel H., Haines R., Bradbury P., Stevens A. (2019). Regional protein expression in human Alzheimer’s brain correlates with disease severity. Commun. Biol..

[81] Batkulwar K., Godbole R., Banarjee R., Kassaar O., Williams R.J., Kulkarni M.J. (2018). Advanced glycation end products modulate amyloidogenic APP processing and tau phosphorylation: A mechanistic link between glycation and the development of Alzheimer’s disease. ACS Chem. Neurosci..

[82] Ilic K., Mlinac-Jerkovic K., Jovanov-Milosevic N., Simic G., Habek N., Bogdanovic N., Kalanj-Bognar S. (2019). Hippocampal expression of cell-adhesion glycoprotein neuroplastin is altered in Alzheimer’s disease. J. Cell Mol. Med..

[83] Lue L.F., Schmitz C.T., Serrano G., Sue L.I., Beach T.G., Walker D.G. (2015). TREM2 protein expression changes correlate with Alzheimer’s disease neurodegenerative pathologies in post-mortem temporal cortices. Brain Pathol..

[84] Bekris L.M., Galloway N.M., Montine T.J., Schellenberg G.D., Yu C.E. (2010). APOE mRNA and protein expression in postmortem brain are modulated by an extended haplotype structure. Am. J. Med. Genet. Part B Neuropsychiatr. Genet..

[85] Caušević M., Farooq U., Lovestone S., Killick R. (2010). β-Amyloid precursor protein and tau protein levels are differently regulated in human cerebellum compared to brain regions vulnerable to Alzheimer’s type neurodegeneration. NeuroSci. Lett..

[86] Campanari M.L., Navarrete F., Ginsberg S.D., Manzanares J., Sáez-Valero J., García-Ayllón M.S. (2016). Increased expression of readthrough acetylcholinesterase variants in the brains of Alzheimer’s disease patients. J. Alzheimers Dis..

[87] Bartolotti N., Bennett D.A., Lazarov O. (2016). Reduced pCREB in Alzheimer’s disease prefrontal cortex is reflected in peripheral blood mononuclear cells. Mol. Psychiatry.

[88] Jin N., Qian W., Yin X., Zhang L., Iqbal K., Grundke-Iqbal I., Gong C.X., Liu F. (2013). CREB regulates the expression of neuronal glucose transporter 3: A possible mechanism related to impaired brain glucose uptake in Alzheimer’s disease. Nucleic Acids Res..

[89] Gu Q., Cuevas E., Raymick J., Kanungo J., Sarkar S. (2020). Downregulation of 14-3-3 Proteins in Alzheimer’s disease. Mol. Neurobiol..

[90] Ginsberg S.D., Mufson E.J., Counts S.E., Wuu J., Alldred M.J., Nixon R.A., Che S. (2010). Regional selectivity of rab5 and rab7 protein upregulation in mild cognitive impairment and Alzheimer’s disease. J. Alzheimers Dis..

[91] Wang S., Wang R., Chen L., Bennett D.A., Dickson D.W., Wang D.S. (2010). Expression and functional profiling of neprilysin, insulin-degrading enzyme, and endothelin-converting enzyme in prospectively studied elderly and Alzheimer’s brain. J. Neurochem..

[92] Sengupta U., Montalbano M., McAllen S., Minuesa G., Kharas M., Kayed R. (2018). Formation of toxic oligomeric assemblies of RNA-binding protein: Musashi in Alzheimer’s disease. Acta Neuropathol. Commun..

[93] Liao Y., Qi X.L., Cao Y., Yu W.F., Ravid R., Winblad B., Pei J.J., Guan Z.Z. (2016). Elevations in the levels of NF-κB and inflammatory chemotactic factors in the brains with Alzheimer’s disease—One mechanism may involve α3 nicotinic acetylcholine receptor. Curr. Alzheimer Res..

[94] Kondratova A.A., Kondratov R.V. (2012). The circadian clock and pathology of the ageing brain. Nat. Rev. Neurosci..

[95] Yamazaki S., Numano R., Abe M., Hida A., Takahashi R., Ueda M., Block G.D., Sakaki Y., Menaker M., Tei H. (2000). Resetting central and peripheral circadian oscillators in transgenic rats. Science.

[96] Buhr E.D., Takahashi J.S. (2013). Molecular components of the Mammalian circadian clock. Circadian Clocks.

[97] Cronin P., McCarthy M.J., Lim A.S.P., Salmon D.P., Galasko D., Masliah E., De Jager F.L., Bennett D.A., Desplats P. (2017). Circadian alterations during early stages of Alzheimer’s disease are associated with aberrant cycles of DNA methylation in BMAL1. Alzheimers Dement..

[98] Song H., Moon M. (2015). Aβ-induced degradation of BMAL1 and CBP leads to circadian rhythm disruption in Alzheimer’s disease. Mol. Neurodegener..

[99] Belanger V., Picard N., Cermakian N. (2006). The circadian regulation of *Presenilin-2* gene expression. Chronobiol. Int..

[100] Chiou Y.Y., Yang Y., Rashid N., Ye R., Selby C.P., Sancar A. (2016). Mammalian period represses and de-represses transcription by displacing CLOCK-BMAL1 from promoters in a Cryptochrome-dependent manner. Proc. Natl. Acad. Sci. USA.

[101] Yang Y., Xu T., Zhang Y., Qin X. (2017). Molecular basis for the regulation of the circadian clock kinases CK1δ and CK1ε. Cell Signal..

[102] Zhou L., Bryant C.D., Loudon A., Palmer A.A., Vitaterna M.H., Turek F.W. (2014). The circadian clock gene Csnk1e regulates rapid eye movement sleep amount, and nonrapid eye movement sleep architecture in mice. Sleep.

[103] Walton K.M., Fisher K., Rubitski D., Marconi M., Meng Q.J., Sládek M., Adams J., Bass M., Chandrasekaran R., Butler T. (2009). Selective inhibition of casein kinase 1 epsilon minimally alters circadian clock period. J. Pharmacol. Exp. Ther..

[104] Harvey K.F., Pfleger C.M., Hariharan I.K. (2003). The Drosophila Mst ortholog, hippo, restricts growth and cell proliferation and promotes apoptosis. Cell.

[105] Meng Z., Moroishi T., Guan K.L. (2016). Mechanisms of Hippo pathway regulation. Genes Dev..

[106] Zhao B., Ye X., Yu J., Li L., Li W., Li S., Yu J., Lin J.D., Wang C.Y., Chinnaiyan A.M. (2008). TEAD mediates YAP-dependent gene induction and growth control. Genes Dev..

[107] Praskova M., Khoklatchev A., Ortiz-Vega S., Avruch J. (2004). Regulation of the MST1 kinase by autophosphorylation, by the growth inhibitory proteins, RASSF1 and NORE1, and by Ras. Biochem. J..

[108] Liu Y., Deng J. (2019). Ubiquitination-deubiquitination in the Hippo signaling pathway. Oncol. Rep..

[109] Zhao B., Li L., Tumaneng K., Wang C.Y., Guan K.L. (2010). A coordinated phosphorylation by Lats and CK1 regulates YAP stability through SCF(beta-TRCP). Genes Dev..

[110] Schittek B., Sinnberg T. (2014). Biological functions of casein kinase 1 isoforms and putative roles in tumorigenesis. Mol. Cancer.

[111] Dolek N., Saylisoy S., Ozbabalik D., Adapinar B. (2012). Comparison of hippocampal volume measured using magnetic resonance imaging in Alzheimer’s disease, vascular dementia, mild cognitive impairment and pseudodementia. J. Int. Med. Res..

[112] Boopathy G.T.K., Hong W. (2019). Role of hippo pathway-YAP/TAZ signaling in angiogenesis. Front. Cell Dev. Biol..

[113] Qing J., Liu X., Wu Q., Zhou M., Zhang Y., Mazhar M., Huang X., Wang L., He F. (2020). Hippo/YAP pathway plays a critical role in effect of GDNF against Aβ-induced inflammation in microglial cells. DNA Cell Biol..

[114] Gogia N., Chimata A.V., Deshpande P., Singh A., Singh A. (2021). Hippo signaling: Bridging the gap between cancer and neurodegenerative disorders. Neural Regen. Res..

