# Supplementary material for: Co-Expression Network Analysis of Micro-RNAs and Proteins in the Alzheimer’s Brain: A Systematic Review of Studies in the Last 10 Years
Source: Cells. 2021 Dec 9;10(12):3479. doi: 10.3390/cells10123479 (PMC8699941; doi:10.3390/cells10123479)
Supplement: Supplementary file 1 [file cells-10-03479-s001.zip › cells-1494973-supplementary.pdf]

SUPPLEMENTARY MATERIALS

Co-expression Network Analysis of Micro-RNAs and Proteins in the Alzheimer's Brain: A Systematic Review

Rachel Tasker [1,†], Joseph Rowlands [1,†], Zubair Ahmed [1,2,3], Valentina Di Pietro [1,2,3,*]

[1]Institute of Inflammation and Ageing, University of Birmingham, Edgbaston, Birmingham, B15 2TT, UK; rxt872@student.bham.ac.uk; joe.j.rowlands747@gmail.com; z.ahmed.1@bham.ac.uk; v.dipetro@bham.ac.uk.
[2]Surgical Reconstruction and Microbiology Research Centre, National Institute for Health Research, Queen Elizabeth Hospital, Birmingham, B15 2TH, UK.
[3]Centre for Trauma Sciences Research, University of Birmingham, Edgbaston, Birmingham, B15 2TT, UK.

[†]These authors contributed equally

***Correspondence:** v.dipietro@bham.ac.uk.

Supplementary Materials
Pathway analyses
Figure S1
Table S1
Table S2
Table S3
Table S4

Table S1: Full list of DE-miRNAs in the 28 selected studies. 113 DE-miRNAs were found differentially expressed either by qRT-PCR or RNAseq, in different regions of AD pateint brains (Braak stage> IV). 53 of them were analysed by qRT-PCR and used for the meta-analysis. **Notes:** HC=Hippocampus, TC=Temporal Cortex, MTG=medial Temporal Gyrus, MFG = Medial Frontal Gyrus, FC = Frontal Cortex, CB = Cerebellum, FNC = Frontal Neo Cortex, STG = Superior temporal gyrus, BDE= Brain Derived Exosome, TL=Temporal Lobe, TNC= Temporal Neo Cortex, FNC= Frontal Neo Cortex, BA10= Brodmann Area 10, PFC= Pre Frontal Cortex, TG= Temporal Gyrus, LC= Locus Coeruleus, EC= Entorhinal Cortex, DG= Dentate Gyrus, BA9= Brodmann Area 9.

miRNA	brain region	Braak stage	fold change	type of analysis	Reference
miR-146	TC	all	UP	qRT-PCR	27
miR-132	CB	all	DOWN	qRT-PCR	27
miR-146a	TC	IV	UP	qRT-PCR	27
miR-146a	FC	IV	UP	qRT-PCR	27
miR-146a	CB	IV	UP	qRT-PCR	27
miR-132	CB	IV	DOWN	qRT-PCR	27
miR-132	TC	IV	UP	qRT-PCR	27
miR-9	FC	IV	UP	qRT-PCR	27
miR-9	CB	IV	UP	qRT-PCR	27
miR-9	TC	IV	UP	qRT-PCR	27
miR-346	FC, BA9	VI	DOWN	qRT-PCR	28
miR-16	FC		DOWN	qRT-PCR	33
miR-16	FC		DOWN	qRT-PCR	33
miR-3157-5p	FC, BDE		UP	RNA-seq	34
miR-190a-5p	FC, BDE		UP	RNA-seq	34
miR-548v	FC, BDE		UP	RNA-seq	34
miR-374b-5b	FC, BDE		UP	RNA-seq	34
miR-374c-3p	FC, BDE		UP	RNA-seq	34
miR-4284	FC, BDE		DOWN	RNA-seq	34
miR-132-5p	FC, BDE		DOWN	RNA-seq	34
miR-5001-3p	FC, BDE		DOWN	RNA-seq	34
miR-219a-2-3p	FC, BDE		UP	RNA-seq	34
miR-550a-3p	FC, BDE		UP	RNA-seq	34

miR-550b-2-5p	FC, BDE		UP	RNA-seq	34
miR-17-5p	FC, BDE		DOWN	RNA-seq	34
miR-18a-5p	FC, BDE		DOWN	RNA-seq	34
miR-501-3p	MTG	V-VI	UP	miRNA-seq	43
miR-10a-5p	MTG	V-VI	UP	miRNA-seq	43
miR-320a	MTG	V-VI	DOWN	miRNA-seq	43
miR-28-3p	MTG	V-VI	UP	miRNA-seq	43
miR-30a-3p	MTG	V-VI	DOWN	miRNA-seq	43
miR-941	MFG	V-VI	DOWN	miRNA-seq	43
miR-184	HC	V-VI	DOWN	miRNA-seq	43
miR-34c-3p	HC	V-VI	DOWN	miRNA-seq	43
miR-375	HC	V-VI	DOWN	miRNA-seq	43
miR-132-3p	HC	V-VI	DOWN	miRNA-seq	43
miR-539-5p	MTG	V-VI	DOWN	miRNA-seq	43
miR-132-5p	MTG	V-VI	DOWN	miRNA-seq	43
miR-212-3p	MTG	V-VI	DOWN	miRNA-seq	43
miR-132-3p	MTG	V-VI	DOWN	miRNA-seq	43
miR-212-5p	MTG	V-VI	DOWN	miRNA-seq	43
miR-582-5p	MFG	V-VI	DOWN	miRNA-seq	43
miR-889-3p	MFG	V-VI	DOWN	miRNA-seq	43
miR-212-5p	MFG	V-VI	DOWN	miRNA-seq	43
miR-212-3p	MFG	V-VI	DOWN	miRNA-seq	43
miR-132-3p	MFG	V-VI	DOWN	miRNA-seq	43
miR-132-5p	MFG	V-VI	DOWN	miRNA-seq	43
miR-10a-5p	MTG	V-VI	UP	qRT-PCR	43
miR-28-3p	MTG	V-VI	UP	qRT-PCR	43
miR-184	HC	V-VI	DOWN	qRT-PCR	43
miR-34c-3p	HC	V-VI	DOWN	qRT-PCR	43
miR-375	HC	V-VI	DOWN	qRT-PCR	43
miR-132-3p	HC	V-VI	DOWN	qRT-PCR	43
miR-132-5p	HC	V-VI	DOWN	qRT-PCR	43
miR-212-3p	HC	V-VI	DOWN	qRT-PCR	43
miR-212-5p	HC	V-VI	DOWN	qRT-PCR	43
miR-539-5p	MTG	V-VI	DOWN	qRT-PCR	43
miR-132-3p	MTG	V-VI	DOWN	qRT-PCR	43
miR-132-5p	MTG	V-VI	DOWN	qRT-PCR	43
miR-212-3p	MTG	V-VI	UP	qRT-PCR	43
miR-212-5p	MTG	V-VI	DOWN	qRT-PCR	43
miR-132-3p	MFG	V-VI	DOWN	qRT-PCR	43
miR-132-5p	MFG	V-VI	DOWN	qRT-PCR	43
miR-212-3p	MFG	V-VI	DOWN	qRT-PCR	43
miR-212-5p	MFG	V-VI	DOWN	qRT-PCR	43
miR-184	HC	V-VI	DOWN	qRT-PCR	43
miR-34c-3p	HC	V-VI	DOWN	qRT-PCR	43
miR-375	HC	V-VI	DOWN	qRT-PCR	43
miR-132-3p	HC	V-VI	DOWN	qRT-PCR	43

miR-132-5p	HC	V-VI	DOWN	qRT-PCR	43
miR-212-3p	HC	V-VI	DOWN	qRT-PCR	43
miR-212-5p	HC	V-VI	DOWN	qRT-PCR	43
miR-486-3p	FC, BDE		UP	RNA-seq	44
miR-200b-5p	FC, BDE		UP	RNA-seq	44
miR-483-5p	FC, BDE		UP	RNA-seq	44
miR-2277-3p	FC, BDE		UP	RNA-seq	44
miR-6858-5p	FC, BDE		UP	RNA-seq	44
miR-141-3p	FC, BDE		UP	RNA-seq	44
miR-3607-3p	FC, BDE		UP	RNA-seq	44
miR-520g-3p	FC, BDE		UP	RNA-seq	44
miR-517b-3p	FC, BDE		UP	RNA-seq	44
miR-483-3p	FC, BDE		UP	RNA-seq	44
miR1287-3p	FC, BDE		UP	RNA-seq	44
miR-4661-5p	FC, BDE		UP	RNA-seq	44
miR-424-3p	FC, BDE		UP	RNA-seq	44
miR-193a-5p	FC, BDE		UP	RNA-seq	44
let-7d-3p	FC, BDE		UP	RNA-seq	44
miR-584-5p	FC, BDE		UP	RNA-seq	44
miR-204-3p	FC, BDE		UP	RNA-seq	44
miR-320a	FC, BDE		UP	RNA-seq	44
miR-16-2-3p	FC, BDE		UP	RNA-seq	44
miR-26b-3p	FC, BDE		UP	RNA-seq	44
miR-34c-3p	FC, BDE		UP	RNA-seq	44
miR-199a-3p	FC, BDE		UP	RNA-seq	44
miR-200c-3p	FC, BDE		UP	RNA-seq	44
miR-23a-3p	FC, BDE		UP	RNA-seq	44
let-7b-3p	FC, BDE		UP	RNA-seq	44
miR-298	TL		DOWN	qRT-PCR	45
miR-128a	TNC		DOWN	qRT-PCR	46
miR-128b	TNC		DOWN	qRT-PCR	46
miR-128b	FNC		DOWN	qRT-PCR	46
miR-15b	FC		DOWN	qRT-PCR	47
miR-132-3p			DOWN	qRT-PCR	48
miR-100			DOWN	qRT-PCR	48
miR-30e-3p			DOWN	qRT-PCR	49
miR-365b-5p			DOWN	qRT-PCR	49
miR-664-3p			DOWN	qRT-PCR	49
miR-1202			DOWN	qRT-PCR	49
miR-4286			DOWN	qRT-PCR	49
miR-4410			DOWN	qRT-PCR	49
miR-455-3p	FC, BA10		UP	qRT-PCR	50
miR-4674	FC, BA10	VI	UP	qRT-PCR	51
miR-6722	FC, BA10	VI	UP	qRT-PCR	51
miR-455-3p	FC, BA10	V	UP	qRT-PCR	51
miR-3613-3p	FC, BA10	V	UP	qRT-PCR	51

miR-122-5p	FC, BA10		DOWN	qRT-PCR	51
miR-132-3p	HC		DOWN	RNA-seq	52
miR-128	HC		DOWN	RNA-seq	52
miR-23a-3p	HC		UP	RNA-seq	52
miR-455-5p	HC		UP	RNA-seq	52
miR-129-5p	HC		DOWN	RNA-seq	52
miR-362-3p	HC		UP	RNA-seq	52
miR-27a-3p	HC		UP	RNA-seq	52
miR-370	HC		DOWN	RNA-seq	52
miR-487b	HC		DOWN	RNA-seq	52
let-7f-5p	HC		UP	RNA-seq	52
miR-223-3p	HC		UP	RNA-seq	52
miR-433	HC		DOWN	RNA-seq	52
miR-195-5p	HC		UP	RNA-seq	52
miR-138-5p	HC		DOWN	RNA-seq	52
miR-142-3p	HC		UP	RNA-seq	52
miR-129-2-3p	HC		DOWN	RNA-seq	52
miR-150-5p	HC		UP	RNA-seq	52
miR-136-5p	HC		DOWN	RNA-seq	52
let-7i-5p	HC		UP	RNA-seq	52
miR-124-3p	HC		DOWN	RNA-seq	52
miR-362-3p	HC		UP	RNA-seq	52
miR-92b-3p	HC		UP	RNA-seq	52
miR-127-3p	HC		UP	RNA-seq	52
miR-329	HC		DOWN	RNA-seq	52
miR-495-3p	HC		DOWN	RNA-seq	52
miR-409-5p	HC		DOWN	RNA-seq	52
miR-487a	HC		DOWN	RNA-seq	52
miR-410	HC		DOWN	RNA-seq	52
miR-543	HC		DOWN	RNA-seq	52
miR-199a-3p	HC		UP	RNA-seq	52
miR-199b-3p	HC		UP	RNA-seq	52
miR-769-5p	HC		DOWN	RNA-seq	52
miR-219-2-3p	HC		DOWN	RNA-seq	52
miR-425-5p	HC		DOWN	RNA-seq	52
miR-200a-3p	HC		UP	RNA-seq	52
miR-92b-3p	HC		DOWN	qRT-PCR	52
miR-129-2-3p	HC		DOWN	qRT-PCR	52
miR-129-5p	HC		DOWN	qRT-PCR	52
miR-132-3p	HC		DOWN	qRT-PCR	52
miR-136-5p	HC		DOWN	qRT-PCR	52
miR-370	HC		DOWN	qRT-PCR	52
miR-409-5p	HC		DOWN	qRT-PCR	52
miR-487a	HC		DOWN	qRT-PCR	52
miR-129-5p	PFC		DOWN	qRT-PCR	52
miR-132-3p	PFC		DOWN	qRT-PCR	52

miR-136-5p	PFC		DOWN	qRT-PCR	52
miR-132-3p	TG		DOWN	qRT-PCR	52
miR-29c	FC		DOWN	qRT-PCR	53
miR-219-5p	FC	IV-V	DOWN	qRT-PCR	54
miR-132	TL		UP	qRT-PCR	55
miR-27a-3p	LC	III-IV	UP	qRT-PCR	56
miR-124-3p	LC	III-IV	UP	qRT-PCR	56
miR-143-3p	LC	III-IV	UP	qRT-PCR	56
miR-143-3p	EC	III-IV	UP	qRT-PCR	56
miR-16	HC	III-IV	UP	qRT-PCR	56
miR-16	HC	VI	DOWN	qRT-PCR	56
miR-34c	HC	III-IV	UP	qRT-PCR	56
miR-107	HC	VI	DOWN	qRT-PCR	56
miR-128a	HC	VI	DOWN	qRT-PCR	56
miR-146a	HC	III-IV	UP	qRT-PCR	56
miR-146a	HC	VI	DOWN	qRT-PCR	56
miR-103	TC		DOWN	qRT-PCR	57
miR-107	TC		DOWN	qRT-PCR	57
miR-15b	TC		DOWN	qRT-PCR	57
miR-16	TC		DOWN	qRT-PCR	57
miR-195	TC		DOWN	qRT-PCR	57
miR-103	HC		DOWN	qRT-PCR	57
miR-107	HC		DOWN	qRT-PCR	57
miR-15b	HC		DOWN	qRT-PCR	57
miR-16	HC		DOWN	qRT-PCR	57
miR-195	HC		DOWN	qRT-PCR	57
miR-132-3p	TC		DOWN	qRT-PCR	59
miR-212-3p	TC		DOWN	qRT-PCR	59
miR-132-3p	PFC		DOWN	qRT-PCR	59
miR-212-3p	PFC		DOWN	qRT-PCR	59
miR-132-3p	PFC		DOWN	qRT-PCR	59
miR-212-3p	PFC		DOWN	qRT-PCR	59
miR-338-5p	HC		DOWN	qRT-PCR	60
miR-219-5p	BA9		DOWN	qRT-PCR	61
miR-124	HC		UP	qRT-PCR	62
miR-124	TC		UP	qRT-PCR	62
miR-132	TC	III-IV	DOWN	qRT-PCR	63
miR-132	TC	VI	DOWN	qRT-PCR	63
miR-212	TC	VI	UP	qRT-PCR	63
miR-425-5p	FC		UP	qRT-PCR	64
miR-603	HC		UP	qRT-PCR	65
miR-7	H, TL		UP	qRT-PCR	66
miR-146a	H, TL		UP	qRT-PCR	66
miR-155	H, TL		UP	qRT-PCR	66
miR-34a	HC		UP	qRT-PCR	67
miR-125b	HC		UP	qRT-PCR	67

miR-146a	HC		UP	qRT-PCR	67
miR-155	HC		UP	qRT-PCR	67

Analysis of MiRNA Targets through DIANA Pathway Analysis

DIANA is the most extensive bioinformatics tool used for miRNA pathway analysis. The database applies algorithms to assess the biological significance of miRNA associations and predict their function via experimentally validated data in TarBase or in silico predicted targets in microT-CDS. This study aims to identify novel pathways so the microT-CDS database, which predicts miRNA targets in the 3′UTR and coding sequences of genes without experimental validation, was utilised. The 113 miRNAs identified either by qRT-PCR or RNAseq in the selected 28 studies were individually uploaded to DIANA and a superset of gene targets was identified. Where selected genes unite on a particular Kyoto Encyclopaedia of Genes and Genomes (KEGG) pathway with ($p<0.05$), this was noted as statistically significant and potential role for miRNA regulation in AD were proposed.

All miRNA identified through the literature search checked for their unique identifiers through MirBase. The following are the miRNA identifiers that were entered into the DIANA database for KEGG pathway analysis: let-7b-3p, let-7d-3p, let-7f-5p, let-7i-5p, miR-100, miR-103, miR-107, miR-10a-5p, miR-1202, miR-122-5p, miR-124-3p, miR-125b, miR-127-3p, miR-128, miR-128a, miR-128b, miR-129-2-3p, miR-129-5p, miR-132-3p, miR-132-5p, miR-136-5p, miR-138-5p, miR-141-3p, miR-142-3p, miR-143-3p, miR-146, miR-146a, miR-150-5p, miR-155, miR-15b, miR-16, miR-16-2-3p, miR-17-5p, miR-184 , miR-18a-5p, miR-190a-5p, miR-193a-5p, miR-195-5p, miR-199a-3p, miR-199b-3p, miR-200a-3p, miR-200b-5p, miR-200c-3p, miR-204-3p, miR-212-3p, miR-212-5p, miR-219-2-3p, miR-219-5p, miR-219a-2-3p, miR-223-3p, miR-2277-3p, miR-23a-3p, miR-26b-3p, miR-27a-3p, miR-27a-3p, miR-28-3p, miR-298, miR-29c, miR-30a-3p, miR-30e-3p, miR-3157-5p, miR-320a, miR-329, miR-338-5p, miR-346, miR-34a, miR-34c-3p, miR-3607-3p, miR-3613-3p, miR-362-3p, miR-365b-5p, miR-370, miR-374b-5b, miR-374c-3p, miR-375, miR-409-5p, miR-410, miR-424-3p, miR-425-5p, miR-4284, miR-433, miR-4410, miR-455-3p, miR-4661-5p, miR-4674, miR-483-3p, miR-483-5p, miR-486-3p, miR-487a, miR-487b, miR-495-3p, miR-5001-3p, miR-501-3p, miR-517b-3p, miR-520g-3p, miR-539-5p, miR-539-5p, miR-543, miR-548v, miR-550a-3p, miR-550b-2-5p, miR-582-5p, miR-584-5p, miR-603, miR-664-3p, miR-6722, miR-6858-5p, miR-7, miR-769-5p, miR-889-3p, miR-9, miR-92b-3p, miR-941.

Analysis of Proteins through DAVID Pathway Analysis

From the 26 included papers, a total of 169 different proteins were found to be differentially expressed in AD. These proteins are listed here: ACHE, AGER, AGT, AHNAK, AIF1, ALAD, AMY1A, ANXA5, APP, AQP4, ARNTL, ASAH1, BACE1, BAG3, BCR, C3, CASP3, CCL2, CCR1, CHGA, CLOCK, CLU, CP, CSNK1E, CTSB, DBI, DKK3, ESD, FGA, FGB, FGG, GJA1, H3F3A, HDGF, HIST1H1C, HIST1H1E, HP, HPX, HSPA1A, HSPB1, IGHA1, IGHG1, IGKC, ISYNA1, ITIH4, LGMN, MAOB, MAP4, MAPT, MARCKS, MECP2, MSI1, MSI2, NAMPT, NFKB1, NPTN, NUCKS1, ORM1, PADI2, PAICS, PBXIP1, PCBD1, PLIN3, PNPO, PRDX1, PRDX6, RAB5A, RAB7A, RBM15B, RIDA, S100A1, S100A11, S100A6, S100A9, SAA1, SELENBP1, SERPINA1, SERPINA3, SERPING1, SOCS4, SOCS7, SPR, STOM, TARDBP, TPD52L1, TREM2, TYROBP, ABCB1, ACTN2, ADAP1, AP1G1, APOE, BIN1, CADPS, CAP2, CIRBP, CORO1A, CORO2B, CRAT, CREB1, CREBBP, DLAT, DLG4, DNAJC6, DNM3, DUSP3, EEF1A1, EEF1B2, EP300, FARSB, GAS7, GLS, GRPEL1, HGS, HOMER1, HSPA4L, IARS2, IDE, IDH3G, IPO7, KIAA0513, KIF5C, LONP1, LRPAP1, LRPPRC, LZTFL1, MAPRE3, METTL3, MME, NDUFA10, NECAB1, OAT, OGDH, OGDHL, OTUB1, OXCT1, PAFAH1B1, PDHX, PDIA3, PHYHIPL, PPME1, PPP2R1A, PREP, PRKRA, PTPA, RAP1GDS1, RGS7, RPH3A, SARS2, SCAI, SDR39U1, SGTB, SH3GL1, SLC2A3, SLIRP, SMS, SNAP25, STXBP1, STXBP3, SUCLA2, SUCLG1, TIMM44, TLN2, TRAP1, VPS35, YARS, YWHAG, YWHAH, YWHAQ

The identified DE-proteins were uploaded to the DAVID database to perform enrichment analysis and pathway identification. Within the DAVID database, the KEGG pathway maps were used to identify the protein pathways that the uploaded DE protein list was associated with. All proteins were uploaded as a gene list under the species "Homo sapiens". Following the pathway tab in the annotation summary results, the KEGG_PATHWAY chart was selected. Despite all 169 protein IDs being recognised only 99 were associated with KEGG pathways that yielded a total of 164 different KEGG pathways. To determine which pathways were the most significant, functional enrichment analysis was performed using a minimum gene count ≥ 3 and a maximum EASE score of 0.1.

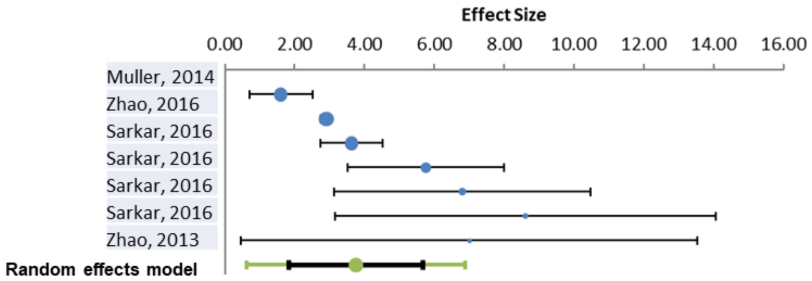

Effect size	95% - CI Lower limit	Upper limit	Weight
1.60	0.07	2.50	23.19%
2.90	0.40	3.06	26.64%
3.63	1.03	4.51	23.19%
5.75	1.69	7.99	13.25%
6.80	2.50	10.48	7.14%
8.60	3.00	14.05	3.82%
7.00	5.00	13.54	2.77%

Heterogeneity: $I^2 = 84.49\%$, $T^2 = 1.41$, $p = 0.0001$

Figure S1. Meta analysis for miR-146a (upregulated miRs).

Table S2A. AXIS quality appraisal tool results for miRNA studies.

	Intro		Methods								Results			Discussion		Other		Total
	1) were the aims/ objectives of the study clear?	2) was the study design appropriate for the stated aim(s)	3) was the sample size of the groups justified?	4) was the target/reference population clearly defined? / is it clear who the research was about?	5) was the sample frame taken from an appropriate population base so that it closely represented the target/reference population under investigation?	6) Was the selection process likely to select subjects/participants that were representative of the target reference population under investigation?	7) were risk factor and outcome variables measures appropriate to the aims of the study?	8) were the risk factor and outcome variables measured correctly using instruments/measurements that had been trialled, piloted or published previously?	9) is it clear what was used to determine statistical significance and/or precision estimates?	10) were the methods (including statistical methods) sufficiently described to enable them to be repeated?	11) were the basic data adequately described?	12) Were the results internally consistent?	13) were the results presented for all the analyses described in the method?	14) were the authors discussions and conclusions justified by the results?	15) were the limitations of the study discussed?	16) were there any funding sources or conflicts of interest that may affect the authors interpretation of the results?	17) was ethical approval or consent of participants	Overall Score (%)
Annese *et al*. 2020 (43)	+	+	-	+	+	+	+	+	+	+	+	+	+	+	+	+	+	**94**
Cheng *et al*. 2020 (44)	+	+	-	+	+	-	+	+	+	+	+	+	+	+	+	+	+	**94**
Chopra *et al*. 2020 (45)	+	+	-	+	+	+	+	+	+	+	-	+	+	+	+	+	+	**88**
Culpan *et al*. 2012 (46)	+	+	-	+	+	-	+	+	+	-	+	+	+	-	+	+	+	**88**
Gong *et al*. 2017 (47)	+	+	-	+	-	-	+	+	+	-	+	+	+	+	-	+	+	**82**
Henriques *et al*. 2020 (48)	+	+	-	+	+	-	+	+	+	+	+	+	+	+	+	+	+	**94**
Herbert *et al*. 2013 (49)	+	+	-	+	+	+	+	+	-	-	+	+	+	+	+	+	+	**82**
Kumar *et al*. 2017 (51)	+	+	-	+	+	-	+	+	+	+	+	+	+	+	+	+	+	**94**
Kumar *et al*. 2018 (50)	+	+	+	+	+	+	+	+	+	+	+	+	+	+	+	+	+	**100**
Lau *et al*. 2013 (52)	+	+	+	+	-	+	+	+	+	+	+	+	+	+	+	+	+	**100**

Lei *et al*. 2015 (53)	+	+	-	+	+	+	+	+	+	+	+	+	+	+	-	+	+	**88**
Li *et al*. 2019 (54)	+	+	-	+	+	-	+	+	+	+	+	+	+	+	-	+	+	**82**
Liu *et al*. 2019 (55)	+	+	-	+	-	-	+	+	+	+	+	+	+	+	-	+	+	**76**
Llorens *et al*. 2017 (56)	+	+	-	+	+	-	+	+	+	+	+	+	+	+	-	+	+	**82**
Long *et al*. 2019 (28)	+	+	+	+	+	+	+	+	+	+	+	+	+	+	+	-	+	**94**
Moncini *et al*. 2016 (57)	+	+	-	+	+	+	+	+	+	+	+	+	+	+	-	+	+	**88**
Muller *et al*. 2014 (59)	+	+	-	+	+	+	+	+	+	+	-	+	+	+	+	+	+	**88**
Pichler *et al*. 2017 (59)	+	+	+	+	+	+	+	+	+	+	-	+	+	+	-	+	+	**88**
Qian *et al*. 2019 (60)	+	+	-	+	+	+	+	+	+	+	+	+	+	+	-	+	+	**88**
Santa-Maria *et al*. 2015 (61)	+	+	-	+	+	+	+	+	+	+	+	+	+	+	+	+	+	**92**
Sarkar *et al*. 2016 (27)	+	+	-	+	+	+	+	+	+	+	+	+	+	+	-	+	+	**88**
Wang *et al*. 2018 (62)	+	+	-	+	-	-	+	+	+	+	+	+	+	+	-	+	+	**71**
Wong *et al*. 2013 (63)	+	+	-	+	+	-	+	+	+	+	+	+	+	+	-	+	+	**88**
Yuan *et al*. 2020 (64)	+	+	-	+	+	-	+	+	+	+	+	+	+	+	-	+	+	**82**
Zhang *et al*. 2016 (65)	-	+	-	+	+	-	+	+	+	+	+	+	+	+	+	+	+	**82**
Zhao *et al*. 2013 (66)	+	+	-	+	-	-	+	+	+	+	+	-	+	+	-	+	+	**82**
Zhao et al. 2016 (67)	+	+	-	+	+	+	+	+	+	+	+	+	+	+	-	+	+	**88**
Zhong et al. 2018 (33)	+	+	-	+	+	-	+	+	+	+	+	+	+	+	-	+	+	**82**
**Notes:** - =YES, + = NO, X = Not Applicable																		

Table S2B. AXIS quality appraisal tool results for protein studies.

	**Intro**		**Methods**								**Results**			**Discussion**		**Other**		**Total**
	1) were the aims/ objectives of the study clear?	2) was the study design appropriate for the stated aim(s)	-3) was the sample size of the groups justified?	4) was the target/reference population clearly defined? / is it clear who the research was about?	5) was the sample frame taken from an appropriate population base so that it closely represented the target/reference population under investigation?	6) Was the selection process likely to select subjects/participants that were representative of the target reference population under investigation?	7) were risk factor and outcome variables measures appropriate to the aims of the study?	8) were the risk factor and outcome variables measured correctly using instruments/measurements that had been trialled, piloted or published previously?	9) is it clear what was used to determine statistical significance and/or precision estimates?	10) were the methods (including statistical methods) sufficiently described to enable them to be repeated?	11) were the basic data adequately described?	12) Were the results internally consistent?	13) were the results presented for all the analyses described in the method?	14) were the authors discussions and conclusions justified by the results?	15) were the limitations of the study discussed?	16) were there any funding sources or conflicts of interest that may affect the authors interpretation of the results?	17) was ethical approval or consent of participants attained?	Score (%)
Beckelman *et al*. 2016 (68)	+	+	-	+	+	+	+	+	+	+	+	+	+	+	-	-	+	**82**
Chiu *et al*. 2015 (69)	+	+	+	+	+	+	+	+	+	+	+	+	+	+	+	+	+	**100**
Shepherd *et al*. 2020 (70)	+	+	-	+	+	+	+	+	+	+	+	+	+	+	-	-	+	**82**
Chen *et al*. 2012 (71)	-	+	-	+	+	+	+	+	-	-	+	+	+	+	-	-	+	**65**
Holler *et al*. 2014 (72)	+	+	-	+	+	+	+	+	+	+	+	+	+	+	-	+	+	**88**
Walker *et al*. 2015 (73)	+	+	-	+	+	+	+	+	+	+	+	+	+	+	-	+	+	**88**
Glennon *et al*. 2013 (74)	-	+	-	+	+	+	+	+	+	+	+	+	+	+	-	+	+	**82**

Byman *et al*. 2018 (75)	+	+	-	+	+	+	+	+	+	+	+	+	-	+	+	-	+	**82**
Huang *et al*. 2020 (76)	+	+	-	+	+	+	+	+	+	+	+	+	+	+	+	X	+	**88**
Yoo *et al*. 2020 (77)	+	+	-	+	+	+	+	+	+	+	+	+	+	+	-	+	+	**88**
Chen *et al*. 2012 (78)	+	+	-	+	+	+	+	+	-	-	+	+	+	+	+	+	+	**82**
Gu *et al*. 2020 (79)	+	+	-	+	+	+	+	+	+	+	+	+	+	+	-	+	+	**88**
Xu *et al*. 2019 (80)	+	+	-	+	+	+	+	+	-	-	+	+	+	+	-	+	+	**76**
Batkulwar *et al*. 2018 (81)	+	+	-	+	+	+	+	+	+	+	+	+	+	+	-	+	+	**88**
Ilic *et al*. 2019 (82)	+	+	-	+	+	+	+	+	+	+	+	+	+	+	-	+	+	**88**
Lue *et al*. 2015 (83)	+	+	-	+	+	+	+	+	+	+	+	+	+	+	-	+	+	**88**
Bekris *et al*. 2010 (84)	+	+	+	+	+	+	+	+	+	+	+	+	+	+	+	+	+	**100**
Causevic *et al*. 2010 (85)	+	+	-	+	+	+	+	+	+	+	+	+	-	+	+	X	+	**82**
Campanari *et al*. 2016 (86)	-	+	-	+	+	+	+	+	+	+	+	+	+	+	-	+	+	**82**
Bartolotti *et al*. 2016 (87)	+	+	-	+	+	+	+	+	-	-	+	+	+	+	+	+	+	**82**
Jin *et al*. 2013 (88)	-	+	-	+	+	+	+	+	+	+	+	+	+	+	-	+	+	**82**
Gu *et al*. 2020 (89)	+	+	-	+	+	+	+	+	-	-	+	+	+	+	-	-	+	**71**
Ginsberg *et al*. 2010 (90)	+	+	-	+	+	+	+	+	+	+	+	+	+	+	-	+	+	**88**
Wang *et al*. 2010 (91)	-	+	-	+	+	+	+	+	+	+	+	+	+	+	-	+	+	**82**
Sengupta *et al*. 2018 (93)	-	+	-	+	+	+	+	+	+	+	+	+	+	+	-	+	+	**82**
Liao *et al*. 2016 (93)	+	+	-	+	+	+	+	+	+	+	+	+	+	+	-	-	+	**82**
**Notes:** - =YES, + = NO, X = Not Applicable																		

Table S3A. Risk of bias in miRNA studies

	Bias arising from randomisation process	Bias due to deviations from intended interventions	Bias due to missing data	Bias in management of outcomes	Bias in selection of the reported result	Overall bias
Annese *et al*. 2020 (43)	-	+	+	+	+	+
Cheng *et al*. 2020 (44)	-	+	+	+	+	+
Chopra *et al*. 2020 (45)	-	+	+	+	+	+
Culpan *et al*. 2012 (46)	-	+	+	+	+	+
Gong *et al*. 2017 (47)	-	+	+	+	+	+
Henriques *et al*. 2020 (49)	**X**	+	+	+	+	+
Herbert *et al*. 2013 (48)	-	+	+	+	+	+
Kumar *et al*. 2017 (51)	-	+	+	+	+	+
Kumar *et al*. 2018 (50)	-	+	+	+	+	+
Lau *et al*. 2013 (52)	-	+	+	+	+	+
Lei *et al*. 2015 (53)	-	+	+	+	+	+
Li *et al*. 2019 (54)	-	+	+	+	+	+
Liu *et al*. 2019 (55)	-	+	+	+	+	+
Llorens *et al*. 2017 (56)	-	+	+	+	+	+
Long *et al*. 2019 (28)	+	+	+	+	+	+
Moncini *et al*. 2016 (57)	-	+	+	+	+	+
Muller *et al*. 2014 (58)	-	+	+	+	+	+
Pichler *et al*. 2017 (59)	-	+	+	+	+	+
Qian *et al*. 2019 (60)	-	+	+	+	+	+
Santa-Maria *et al*. 2015 (61)	-	+	+	+	+	+
Sarkar *et al*. 2016 (27)	-	+	+	+	+	+
Wang *et al*. 2018 (62)	-	+	+	+	+	+
Wong *et al*. 2013 (63)	-	+	+	+	+	+
Yuan *et al*. 2020 (64)	-	+	+	+	+	+

Zhang *et al*. 2016 (65)	+	+	+	+	+	+
Zhao *et al*. 2013 (66)	-	+	+	+	+	+
Zhao et al. 2016 (67)	-	+	+	+	**X**	+
Zhong et al. 2018 (33)	-	+	+	+	+	+
**Notes:** - =YES, + = NO, X = Not Applicable						

Table S3B. Risk of bias in miRNA studies

	Bias arising from randomisation process	Bias due to deviations from intended interventions	Bias due to missing outcome data	Bias in measurement of the outcome	Bias in selection of reported results	Overall bias
Beckelman *et al*. 2016 (68)	X	+	+	+	+	+
Chiu *et al*. 2015 (69)	X	+	+	+	+	+
Shepherd *et al*. 2020 (70)	X	+	+	+	+	+
Chen *et al*. 2012 (71)	X	+	+	+	+	+
Holler *et al*. 2014 (72)	X	+	+	+	+	+
Walker *et al*. 2015 (73)	X	+	+	+	+	+
Glennon *et al*. 2013 (74)	X	+	+	+	+	+
Byman *et al*. 2018 (75)	X	+	+	+	+	+
Huang *et al*. 2020 (76)	X	+	+	+	+	+
Yoo *et al*. 2020 (77)	X	+	+	+	+	+
Chen *et al*. 2012 (78)	X	+	+	+	+	+
Gu *et al*. 2020 (79)	X	+	+	+	+	+
Xu *et al*. 2019 (80)	-	+	+	+	+	+
Batkulwar *et al*. 2018 (81)	X	+	+	+	+	+
Ilic *et al*. 2019 (82)	X	+	+	+	+	+
Lue *et al*. 2015 (83)	X	+	+	+	+	+
Bekris *et al*. 2010 (84)	X	+	+	+	+	+
Causevic *et al*. 2010 (85)	X	+	+	+	+	+
Campanari *et al*. 2016 (86)	X	+	+	+	+	+
Bartolotti *et al*. 2016 (87)	-	+	+	+	+	+
Jin *et al*. 2013 (88)	X	+	+	+	+	+
Gu *et al*. 2020 (89)	X	+	+	+	+	+
Ginsberg *et al*. 2010 (90)	-	+	+	+	+	+
Wang *et al*. 2010 (91)	X	+	+	+	+	+
Sengupta *et al*. 2018 (92)	X	+	+	+	+	+
Liao *et al*. 2016 (93)	X	+	+	+	+	+

Notes: - =YES, + = NO, X = Not Applicable

Table S4A.

miRNA	brain region	Braak stage	fold change	SD	no. AD	Age AD	sex AD	no C	age C	sex C	PMI (hrs)	storage	extraction	selection	normalised to:	analysis	reference
miR-132-3p	MFG	v-vi	0.125	0.2	5	-		5	-		C=(42+-14.86) AD=(19.56+-10.27)		TaqMan™ Advanced miRNA cDNA Synthesis Kit (Thermo Fisher Scientific)		-	qRT-PCR assay original cohort	Annese et al 2018
miR-132-3p	MTG	v-vi	0.9	0.3	5	-		5	-							qRT-PCR assay original cohort	Annese et al 2019
miR-132-3p	HC	v-vi	0.3	0.4	9	-	.+4m	9	-	.+4m						qRT-PCR enlarged cohort	Annese et al 2020
miR-132-3p	PFC	v	0.33	0.3	41	-		23	-			frozen in liquid nitrogen.	Trizol® (Invitrogen)		miR-9-5p.	qRT-PCR validation study	Lau et al 2013
miR-132-3p	TG	v	0.35	0.5	41	-		23	-							qRT-PCR validation study	Lau et al 2014
miR-132-3p	HC	v	0.5	0.2	41	-		23	-							qRT-PCR validation study	Lau et al 2015
miR-132-3p	CB	all	0.1	0.01	13	75.6	6m.7f	10	76.5	5m.5f	c=11.3 AD=14.5	frozen	miRNeasy Micro Kit	databases	-	qRT-PCR	Sarkar et al 2016

miR-132-3p	CB	iv	0.1	0.01	13	75.6	6m.7f	10	76.5	5m.5f			isolation kit (Qiagen)		-	qRT-PCR	Sarkar et al 2017
miR-132-3p	TC	vi	0.22	0.2	16	81.7	6m.10f	16	76.5	10m.6f	-		laser capture		miR-9	qPCR	Wong et al 2013
miR-132-3p	-	mixed.	0.35	0.1	8	78.3	5m.3f	71.4	77.9	6m.2f	c=21.8 AD=18.4	snap frozen -80C	Trizol®		miR-16	qRT-PCR validation study	Herbert et al 2013
miR-132-3p	TC	iv	4.42	1.12	13	75.6	6m.7f	10	76.5		c=11.3 AD=14.5		miRNeasy Micro Kit isolation kit (Qiagen)		-	qRT-PCR	Sarkar et al 2017
miR-132-3p	TC	-	**6**	**1.5**	10			10					TRIzol (Invitrogen		-	qRT-PCR	Liu et al 2019

Notes: HC=Hippocampus, TC=Temporal Cortex, MTG=Medial Temporal Gyrus, MFG = Medial Frontal Gyrus FC = Frontal Cortex, CB = Cerebellum, FNC = Frontal Neo Cortex, STG = Superior temporal gyrus, BDE= Brain Derived Exosome

Table S4B.

miRNA	brain region	Braak stage	fold change	SD	analysis	no AD	age AD	sex AD	no C	age C	sex C	PMI (hr)	storage	extraction	selection	reference
miR-146a	HC	vi	0.9	0.04	qRT-PCR	10	77.8	7m.5f	11	82.5	4m.7f	3-6		miRNeasy Mini Kit (Qiagen)		Muller et al 2014
miR-146a	HC	iv	1.6	0.07	qRT-PCR	10	77.8	7m.5f	11	82.5	4m.7f			miRNeasy Mini Kit (Qiagen)		Muller et al 2015
miR-146a	HC, TL	-	2.9	0.4	RT-PCR	12	73.5		3	71	8	2.3		TRIzol (Invitrogen) miRNeasy Mini Kit		ZHao et al 2016
miR-146a	TC	all	3.63	1.03	qRT-PCR	13	75.6	6m.7f	10	76.5	5m.5f	c=11.3 AD=14.5	frozen	miRNeasy Micro Kit isolation kit (Qiagen)	databases	Sarkar et al 2016
miR-146a	TC	iv	5.75	1.69	qRT-PCR	13	75.6	6m.7f	10	76.5	5m.5f	c=11.3 AD=14.6	frozen	miRNeasy Micro Kit isolation kit (Qiagen)	databases	Sarkar et al 2017
miR-146a	CB	iv	6.8	2.5	qRT-PCR	13	75.6	6m.7f	10	76.5	5m.5f	c=11. AD=14.8	frozen	miRNeasy Micro Kit isolation kit (Qiagen)	databases	Sarkar et al 2018
miR-146a	FC	iv	8.6	3	qRT-PCR	13	75.6	6m.7f	10	76.5	5m.5f	c=11. AD=14.7	frozen	miRNeasy Micro Kit isolation kit (Qiagen)	databases	Sarkar et al 2019
miR-146a	HC	-	7	5	qRT-PCR	3	72.1		3	71.8		PMI CvsAD (mean 2.0±1.8 vs. 2.0±1.5 h, P~0.94)		TRIzol (Invitrogen)		Zhao et al 2018

Notes: HC=Hippocampus, TC=Temporal Cortex, MTG=Medial Temporal Gyrus, MFG = Medial Frontal Gyrus FC = Frontal Cortex, CB = Cerebellum, FNC = Frontal Neo Cortex, STG = Superior temporal gyrus, BDE= Brain Derived Exosome

Table S4C.

miRNA	brain region	Braak stage	fold change	SD	analysis	no AD	age AD	sex AD	no C	age C	sex C	PMI (hr)	storage	extraction	selection	reference
miR-16	HC	-	0.3	0.15	RT-qPCR	12	83	7m.5f	12	matched	matched	(PMI) < 25 (mean = 10)				Monici et al 2016
miR-16	TC	-	0.44	0.22	RT-qPCR	12	83	7m.5f	12	matched	matched			TRIzol reagent (Invitrogen)		Monici et al 2017
miR-16	FC	-	0.5	0.1	qRT-PCR	30	80-95	-	20	80-95	-	<4	-	Trizol reagent (Invitrogen)		Zhong et al 2018
miR-16	HC	vi	0.5	0.01	qRT-PCR	10			6							Muller et al 2014
miR-16	FC	-	0.5	0.1	qRT-PCR	30	80-95		20	80-95		<4		Trizol reagent (Invitrogen)		Zhong et al 2018

Notes: HC=Hippocampus, TC=Temporal Cortex, MTG=Medial Temporal Gyrus, MFG = Medial Frontal Gyrus FC = Frontal Cortex, CB = Cerebellum, FNC = Frontal Neo Cortex, STG = Superior temporal gyrus, BDE= Brain Derived Exosome

Table S4D.

miRNA	brain region	braak stage	fold change	SD	normalised	analysis	no AD	age AD	sex AD	no C	age C	sex C	PMI (hr)	storage	extraction	selection	reference
miR-212-3p	MFG		0.12	0.2		qRT-PCR assay original cohort	5	73.75		5	77.25		PMI = C=(42+-14.86) AD=(19.56+-10.27)	frozen	RNase A (Qiagen) Qiagen miRNeasy with the use of TRIzol-LS		Annese et al 2018
miR-212-3p	TC	vi	1	0.2	miR-9	qPCR	16	81.7	6m.10f	16	76.5	10m.6f		formalin fixed	Trizol (Invitrogen) Total Nucleic Acid Isolation Kit		Wong et al 2013
miR-212-3p	MTG		3.5	1		qRT-PCR assay original cohort	5	73.75		5	77.25		PMI = C=(42+-14.86) AD=(19.56+-10.27)	frozen	RNase A (Qiagen) Qiagen miRNeasy with the use of TRIzol-LS		Annese et al 2018

Notes: HC=Hippocampus, TC=Temporal Cortex, MTG=Medial Temporal Gyrus, MFG = Medial Frontal Gyrus FC = Frontal Cortex, CB = Cerebellum, FNC = Frontal Neo Cortex, STG = Superior temporal gyrus, BDE= Brain Derived Exosome
